# Water Deficit and Salinity Stress Reveal Many Specific QTL for Plant Growth and Fruit Quality Traits in Tomato

**DOI:** 10.3389/fpls.2018.00279

**Published:** 2018-03-06

**Authors:** Isidore A. Diouf, Laurent Derivot, Frédérique Bitton, Laura Pascual, Mathilde Causse

**Affiliations:** ^1^INRA, UR1052, Génétique et Amélioration des Fruits et Légumes, Centre de Recherche PACA, Montfavet, France; ^2^GAUTIER Semences, Eyragues, France

**Keywords:** tomato, fruit quality, MAGIC population, genotype by environment interaction, QTL mapping

## Abstract

Quality is a key trait in plant breeding, especially for fruit and vegetables. Quality involves several polygenic components, often influenced by environmental conditions with variable levels of genotype × environment interaction that must be considered in breeding strategies aiming to improve quality. In order to assess the impact of water deficit and salinity on tomato fruit quality, we evaluated a multi-parent advanced generation intercross (MAGIC) tomato population in contrasted environmental conditions over 2 years, one year in control vs. drought condition and the other in control vs. salt condition. Overall 250 individual lines from the MAGIC population—derived from eight parental lines covering a large diversity in cultivated tomato—were used to identify QTL in both experiments for fruit quality and yield component traits (fruit weight, number of fruit, Soluble Solid Content, firmness), phenology traits (time to flower and ripe) and a vegetative trait, leaf length. All the traits showed a large genotype variation (33–86% of total phenotypic variation) in both experiments and high heritability whatever the year or treatment. Significant genotype × treatment interactions were detected for five of the seven traits over the 2 years of experiments. QTL were mapped using 1,345 SNP markers. A total of 54 QTL were found among which 15 revealed genotype × environment interactions and 65% (35 QTL) were treatment specific. Confidence intervals of the QTL were projected on the genome physical map and allowed identifying regions carrying QTL co-localizations, suggesting pleiotropic regulation. We then applied a strategy for candidate gene detection based on the high resolution mapping offered by the MAGIC population, the allelic effect of each parental line at the QTL and the sequence information of the eight parental lines.

## Introduction

Abiotic stress is one of the main factors limiting crop productivity and yield in agriculture. It occurs when plants experience any fluctuation in the growing habitat that alters or disrupts their metabolic homeostasis (de Oliveira et al., 2013). Among the abiotic stresses, drought and salinity are the most common threatening global food demand. Their adverse effect on agriculture is expected to increase with the predicted climate change (Dai, 2011; Shrivastava and Kumar, 2015). Both drought and salinity stresses drive a series of morphological, physiological, and molecular changes in plants that are overall linked to adaptive mechanisms triggered by the plant to survive, or may simply be pathological consequences of stress injury (Zhu, 2002). Indeed, water deficit has several impacts on plant development due to the decrease of the plant's relative water content and water potential. Osmotic stress and limited nutrient uptake are then observed with stomatal closure, reduced photosynthesis activity, oxidative stress, and leaf growth inhibition. These behaviors are well reviewed by Farooq et al. (2012) and Chaves et al. (2003). For saline soil condition, plants are subjected to stress in two phases: a rapid osmotic stress phase starting immediately (due to the concentration of salt outside the roots) and a second ionic phase that starts when the accumulation of salt in the old leaves reaches a toxic level. The osmotic stress triggered by salinity has almost the same effect as drought with photosynthesis limitation, leaf growth inhibition, and ROS accumulation (Munns and Tester, 2008). Plants deploy a variety of adaptive strategies facing drought and salinity, including osmotic adjustment with the accumulation of osmo-protectants compounds, ROS detoxification, stomatal closure, and cellular signaling.

Drought and salinity arise with other adverse environmental factors threatening crop productivity in many species as a consequence of global climate changes. This has led plant breeders to renew their focus on understanding the molecular basis of plant adaptation to environment, in order to maintain high crop yielding by creating new varieties adapted to limited environmental conditions. As noted by Marais et al. (2013), plant responses to adverse conditions can be viewed as phenotypic plasticity (PP) and may lead to GxE when there is a genetic part shaping these responses. Understanding the molecular mechanism entailing PP and GxE is of great relevance in breeding strategies mainly if different growing areas (or cultural conditions) are targeted. For both PP and GxE, different underpinning models were suggested in the literature. PP can be viewed as additive effect of environmentally sensitive loci meaning that the same loci affect the phenotype in a set of environments at variable degrees, or specific regulatory loci altering differently the gene expression, in the different environments (Via et al., 1995). Non-additive effect such as over-dominance and epistasis or epigenetics can also be at the basis of the occurring GxE. El-Soda et al. (2014) present several statistical models to depict GxE into its individual genetic components through the identification of interactive QTL (QTLxE). Considering plasticity as an individual trait, some studies showed that loci linked to PP are in the vast majority also identified as QTLxE (Ungerer et al., 2003; Gutteling et al., 2007; Tétard-Jones et al., 2011).

Cultivated tomato is a crop moderately sensitive to salinity that can tolerate up to 2.5 dS/m EC, with minor negative impact on yield (Scholberg and Locascio, 1999). Caro et al. (1991) have found that small fruit accessions *S. lycopersicum* var *cerasiforme* are less sensitive to salinity than the large fruit group *S. lycopersicum* var *lycopersicum*. For drought, a negative impact on yield is observed from a limitation of water supply by 50% compared to control (well irrigated) (Ripoll et al., 2014; Albert et al., 2016a). Under such stresses, tomato yield components as well as fruit quality are greatly affected with different effects depending on the variety, the stage and duration of stress application and also the interaction with other environmental conditions like temperature, light, or relative humidity (Maas and Hoffman, 1977; Scholberg and Locascio, 1999; Ripoll et al., 2014). Furthermore, the genetic background may strongly modify the response to stress conditions (Albert et al., 2016b). This makes selection of genotypes tolerant to water deficit and salinity with high productivity and fruit quality a challenging task.

Several studies revealed that water deficit (WD) and salinity stress (SS) can improve fruit quality through higher accumulation of quality compounds and anti-oxidant (Mitchell et al., 1991; Du et al., 2008; Huang et al., 2009; Albert et al., 2016a; Ripoll et al., 2016). SS also increases inorganic ion content of salinized plants (Mitchell et al., 1991; Navarro et al., 2005). In many species, particularly for fruit and vegetables, quality is a main objective for variety improvement. Breeding for quality arose with the increasing demand of high quality products from consumers these last decades. Accordingly to its definition (Shewfelt, 1999; Causse et al., 2001), quality is complex and involves several chemical, physical, and organoleptic characteristics that can be directly related to consumer preferences or to the requirement of market-oriented production. Many quantitative trait loci (QTL) related to fruit quality traits were identified in several species (Causse et al., 2001; Monforte et al., 2004; Kenis et al., 2008; Eduardo et al., 2011). These studies revealed that most of the quality components are polygenic and based on multiple correlated traits, some of which being regulated by pleiotropic or linked QTLs (Monforte et al., 2004; Kenis et al., 2008).

Multi-parent populations require crosses between more than two inbred parental lines to generate RIL progeny. They include Nested Association Mapping (called NAM, Yu et al., 2008) or Multi-parent Advanced Generation Inter-cross (called MAGIC) populations (Kover et al., 2009; Huang et al., 2012). The interest of multi-parent populations relies on the mating design allowing more genetic diversity to occur in the offspring, which besides undergoes several recombination events. The first MAGIC population was developed in mouse (Threadgill et al., 2002) and expanded to several plant species (Kover et al., 2009; Huang et al., 2012; Bandillo et al., 2013; Milner et al., 2016). The MAGIC populations have some advantages with respect to association panel for GWAS because of the absence of structure and the balanced allelic frequencies. They already demonstrated their capacity to increase length of genetic maps and detect QTL with reduced confidence intervals compared to bi-parental progenies (Pascual et al., 2015; Gardner et al., 2016). Nevertheless, due to the complexity of the mating design, statistical methods used in bi-parental or GWAS populations are not efficient. A regression model estimating all parental effects was proposed by Huang and George (2011).

In the present study we investigated the effect of salinity stress and water deficit on tomato for quality, yield component, vegetative, and phenology traits, using a MAGIC population based on the cultivated tomato and which underwent several recombination generations. Thus, the objectives were: (1) to assess and compare the impact of both WD and SS at phenotypic level and the trait plasticity, (2) to study the genetic determinants of tomato response to SS and WD and to identify interactive QTL using plasticity and (3) to select candidate genes, based on the parental allelic effect and their genomic sequences.

## Materials and methods

### Plant materials

We analyzed the MAGIC tomato population created at INRA center of Avignon (France). It was derived from the cross of eight parental lines, four of them belonging to the small fruit group *S. lycopersicum* var. *cerasiforme* (Cervil, Criolo, Plovdiv24A, and LA1420) and four lines with large fruit from *S. lycopersicum* var. *lycopersicum* group (Levovil, Stupicke Polni Rane, LA0147 and Ferum). Parent's selection was carefully operated within a core collection of 360 cultivated tomatoes to comprise the maximum diversity, notably the genomes of the four *cerasiforme* accessions representing a mosaic between wild and cultivated tomato genomes. A population of 400 families was obtained following the crossing design detailed in Pascual et al. (2015). The genomes of all parental lines were fully sequenced allowing the identification of about 4 millions of single nucleotide polymorphisms (SNP) (Causse et al., 2013).

### Greenhouse trials

The MAGIC population was grown in contrasted conditions in Morocco (Gautier Semences breeding station) over 2 years in greenhouse with similar experimental procedures. Plants were grown in 5L plastic pots filled with loamy substrate (Klasmann 533) and treatments were applied by row. Stressed and control rows were placed side-by-side, each genotype in the stressed row facing its replicate in the control one. The first year of experiment (Exp.1), water deficit and control (well irrigated) treatment were applied, while the second year (Exp.2) was dedicated to salinity stress and its control treatment. The average temperature and relative humidity in the greenhouses were very similar in both experiment with 20.82°C and 60.68 HR for Exp.1 and 21.74°C and 61.60 HR for Exp.2. However, the management of electrical conductivity (EC) differed between the two experiments. In Exp.1, water supply was reduced for WD treatment with respect to the control treatment where plants were subjected to the optimal irrigation. WD treatment consisted in the reduction of irrigation by 25% at the first flowering truss of Cervil (the earliest parent) and by 50% at the second flowering truss. The EC of the loamy substrate was measured in the pots for each plant with a “GroSens HandHeld” instrument, giving an average value of 1.97 dS/m for the two treatments. In Exp.2, both control and salinity treatments were not restricted in the amount of water intake but differed in the EC application. A fertigation solution with a pH of 6.1 and EC of 3 dS/m was used for both treatments at the beginning of the culture until the 2nd truss flowering of at least half of the plants. Then, salt treatment was enriched with NaCl solution and salinity was evaluated by measuring the EC of the substrate every week. On average, the EC of the substrate was 3.76 dS/m in control treatment and 6.50 dS/m in salinity treatment. The average difference in EC between the controls treatments over the two experiments was thus 1.79 dS/m. First and last rows in the greenhouse were considered as border lines and border genotypes were also placed at the end of rows. The eight parental lines and four F1 hybrids were tested together with 241 MAGIC lines in Exp.1 and 253 MAGIC lines in Exp.2.

### Plant phenotyping

Seven traits were measured in both experiments. For phenology, flowering date (date of first open flower on 4th truss) and maturity date (first ripe fruit on the 4th truss) were recorded. Then time to flower (Flw) and time to ripe (RIP) were recorded as the day number between the sowing date and flowering date for Flw and between the flowering date and maturity date for RIP. Leaf length (Leaf) was measured as vegetative trait for each plant under the 5th truss. Fruits were harvested at maturity every week and for each genotype, fruit number was recorded on plants and fruit weight (FW) measured for at least 10 fruit per genotype on truss 3, 4, 5, and 6. For sugar content in Exp.1, 3 fruits harvested on truss 4 and 5 were pooled and crushed to obtain a fluid on which the soluble solid content (SSC) was measured with an electronic refractometer. In Exp.2 only fruits within each truss were pooled for SSC measurements. A durometer was used to measure fruit firmness (Firm), applying a pressure on the surface of the fruit measuring the strength needed to retract the durometer's tip. Five fruits per genotype were used with two measures per fruit.

For every trait in each experiment, phenotypic plasticity (PP) was measured by the relative difference between the control and stress treatments. For a trait (k) and for a single genotype, we calculated PP as PP_*k*_ = (Stress_*k*_–Control_*k*_)/Control_*k*_ and used these data to identify interactive QTL between stress and control for each experiment. Considering all the genotypes, the average effect of the stress was evaluated in a single experiment by the mean relative variation as (Mean Stress_*k*_–Mean Control_*k*_)/Mean Control_*k*_ and converted in percentage of increase or decrease due to the stress. For convenient comparison between salinity and water deficit effects on phenotypes, the mean variation was also calculated in a second step taking the control in Exp.1 as unique control and all other conditions as stress.

### Statistical analyses

Statistical analyses were performed with the free software R version 3.3.0. Data were firstly checked per trait and per treatment. FW and NFr were log-transformed for normality assumption. Analyses were conducted separately per experiment to allow the comparison of each stress treatment against its control. We tested the fixed effect of genotype and treatment and their interaction by a two way ANOVA following the model: *Yij* = μ + *Gi* + *Tj* + G^*^Tij + ε*ijk*, where Yij represents the phenotype of genotype i (Gi) and treatment j (Tj), G^*^Tij the interaction between genotype and treatment and εijk the residual error. Pearson's correlations were calculated between the mean trait values per treatment and for each trait between treatments within experiment and between the two control treatments. In each treatment, the broad sense heritability (*h*^2^) was evaluated by means of the following ANOVA model where the genotype was considered as random: *Yi* = μ + *Gi* + ε*ij*. Gi and εij are the random effect of genotype and the residual error respectively. The broad sense heritability was then calculated as *h*^2^ = σ^2^G/(σ^2^G+ σ^2^E/r) where σ^2^G and σ^2^E are the genetic and residual variance respectively, and r is the average number of replicates per genotype.

### Haplotype prediction

The MAGIC population is characterized by the complex mating design of the eight parental lines. The parental origin of each allele in the offspring is not intuitive, on the contrary to the bi-parental population. To infer the allelic parental provenance, we estimated the probability of each parent being at the origin of each allele in the MAGIC lines with the function *mpprob* of the mpMap package 2.0 (Huang and George, 2011). We fixed a threshold of 50% above which allelic parental provenance is assigned. These probabilities were further used to perform the QTL identification.

### QTL and QTL×E

The QTL were mapped by interval mapping (IM) procedure with the R package mpMap. Parental probabilities were computed every 2 cM along the genome and at each marker position and then used to estimate parental effects. The regression on the parental allelic effect, at each position where probabilities were computed, allowed the QTL identification. A LOD threshold of 3 was fixed to detect a significant QTL. Confidence interval (CI) of a QTL was estimated with one unit decreasing of the LOD threshold on both sides of a QTL position. Considering one trait, constitutive QTLs were defined when two (or more) QTLs were identified in different conditions (treatments) on the same chromosome with their CI overlapping. They were then considered as a unique QTL expressed in both conditions.

Then, PP was used as single trait for each phenotype, to identify interactive QTL (QTL×E). Before analysis, plasticity data were checked for normality and log transformed for FW and NFr.

### Candidate genes identification

We screened for candidate genes under QTL for the QTL×E and QTL mapped in a CI shorter than 2Mb. For each QTL, we listed the number of polymorphisms and genes present within the CI region based on the sequence information of all parental lines (Causse et al., 2013) and the reference genome (Tomato Genome Consortium, 2012). We filtered the polymorphisms and genes listed in accordance with the parental allelic effects at the QTL. We focused on QTL that present pronounced divergence in the allelic effect of the eight parents, keeping all polymorphisms and genes commonly shared by the parents varying in the same direction and different from those shared by the parents varying in the opposite direction. The putative function of the remaining genes (when annotated) were then checked on the Sol Genomic Network (solgenomics.net) database in order to identify which candidate's annotated function is correlated to the QTL trait of interest.

## Results

### Phenotypic variation in the MAGIC population

The phenotypic variation observed among the MAGIC lines showed transgressions in both directions in comparison to the eight parental values for every trait (Table 1; Supplemental Figure 1). Except FW in control of Exp.2, the highest value in MAGIC lines always exceeded the best parent in every trait by treatment combination.

**Table 1:** Phenotypic variation among MAGIC lines for all traits and treatments.

**Traits**	**Treatments**	**P. range**	**MAGIC lines**			**MV_WD**	**MV_Ctrl2**	**MV_SS**	***h^2^***
			**Min**	**Max**	**Mean**				
SSC	Ctrl1	3.50–7.30	2.80	8.20	5.56	11.78	39.85	75.91	0.72
	WD	4.30–10.10	3.10	10.10	6.21				0.80
	Ctrl2	7.50–9.50	4.00	13.00	7.74	25.96			0.69
	SS	8.50–11.00	6.00	12.5	9.75				0.48
Firm	Ctrl1	50.00–72.00	44.00	73.00	59.58	−2.18	2.02	6.92	0.32
	WD	50.00–68.00	45.50	73.00	58.28				0.09
	Ctrl2	38.50–76.00	36.00	82.00	60.60	4.93			0.64
	SS	57.00–70.00	31.00	84.00	63.58				0.57
FW	Ctrl1	6.88–92.00	10.71	110.00	38.75	−23.05	−38.54	−54.62	0.85
	WD	5.35–95.00	10.54	101.67	29.81				0.83
	Ctrl2	5.00–110.00	5.00	95.00	23.84	−26.52			0.77
	SS	5.00–23.84	2.50	74.28	17.52				0.60
NFr	Ctrl1	6.50–45.50	2.50	105.00	15.61	−15.32	−61.11	−59.85	0.75
	WD	3.00–47.00	3.00	50.00	13.22				0.56
	Ctrl2	2.00–12.50	2.00	23.50	6.20	3.70			0.42
	SS	2.00–15.50	2.00	21.00	6.43				0.40
Leaf	Ctrl1	23.50–42.00	18.00	55.00	32.24	−17.97	−7.92	−16.05	0.85
	WD	23.50–35.50	15.00	48.50	26.45				0.69
	Ctrl2	20.50–35.00	11.00	45.50	29.51	−8.66			0.66
	SS	25.00–31.00	11.50	40.00	26.97				0.58
Flw	Ctrl1	77.50–110.00	77.50	117.00	88.76	−0.77	−10.26	−10.74	0.92
	WD	76.50–107.00	75.50	124.00	88.07				0.92
	Ctrl2	80.50–102.00	75.00	102.00	79.74	−0.60			0.85
	SS	79.00–98.00	74.00	105.00	79.26				0.78
RIP	Ctrl1	51.00–71.50	43.50	74.00	57.88	−2.87	−5.30	−6.59	0.87
	WD	46.50–68.00	44.00	70.00	56.22				0.88
	Ctrl2	46.50–72.00	36.00	79.00	55.72	−0.22			0.64
	SS	47.00–66.00	35.50	75.00	55.59				0.75

*Min, Max and mean are the minimum, maximum and mean values of MAGIC lines. P. range represents the range of the means of the eight parental lines. MV is the relative mean variation, with respect to control in Exp.1 due to treatments under WD (MV_WD), control in Exp.2 (MV_Ctrl2) and salinity (MV_SS). h^2^ is the broad sense heritability calculated for each treatment*.

The comparison of control treatments between the two experiments showed little mean differences for Firm, RIP and Leaf, which had a relative mean variation below 10%. FW, SSC, and NFr varied considerably between the controls by 38.54, 39.85, and 61.11% respectively (Table 1). Statistical analyses were thus conducted separately for each experiment to assess the impact of WD and SS compared to their specific control treatment.

All traits across treatments exhibited heritability above 0.4 except firmness in Exp.1. Heritability ranged from 0.09 for firmness in WD treatment to 0.92 for flowering time in control of Exp.1. In average, Flw, RIP and FW had the highest heritability. For both experiments, heritability varied between control and stress treatment with the highest variation observed for SSC in Exp.2 where *h*^2^ SSC was 0.69 for the control and 0.48 for the salinity treatment. The heritability of a few traits like RIP was poorly impacted by the stress treatments.

The total sum of square of the two way ANOVAs was partitioned in proportion attributed to genotype, treatment and their interaction. A large part of the phenotypic variation was linked to genotype, accounting from 39 to 86% of the total sum of square in Exp.1 and 33 to 72% in Exp.2 (Table 2). Significant effects of treatment were found for every trait in Exp.1 while Exp.2 showed significant treatment effect only for FW, SSC, and Leaf. Similarly, all traits exhibited significant genotype × treatment interaction in both experiments except Firm in Exp.1 and NFr in Exp.2.

**Table 2:** Phenotypic variation attributed to the genotype (G), the treatment (T) and the interaction (GxTreat) effects.

**Traits**	**G**	**SSq G %**	**Treat**	**SSq Treat %**	**GxTreat**	**SSq GxTreat %**	**SSq Resid %**
**EXP.1 (CONTROL vs. WD)**							
Firm	^***^	39.42	^***^	0.86	ns	18.75	40.97
Flw	^***^	86.04	^***^	0.44	^***^	6.48	7.04
FW	^***^	54.16	^***^	9.25	^***^	4.67	31.92
Leaf	^***^	47.75	^***^	14.85	^***^	23.91	13.48
NFr	^***^	55.53	^*^	0.62	^*^	17.88	25.98
RIP	^***^	73.27	^***^	2.77	^***^	13.62	10.34
SSC	^***^	61.75	^***^	6.7	^***^	15.8	15.75
**EXP.2 (CONTROL vs. SS)**							
Flw	^***^	68.76	ns	0.00	^***^	15.4	15.83
FW	^***^	47.14	^***^	6.87	^***^	26.2	19.78
Leaf	^***^	52.36	^***^	3.55	^***^	18.9	25.19
NFr	^***^	42.04	ns	0.18	ns	23.59	34.19
RIP	^***^	59.06	ns	0.01	^*^	17.56	23.36
SSC	^***^	33.45	^***^	27.24	^***^	23.01	16.29

*For each quantitative trait the significance of the explaining factors: G, T and the interaction GxTreat, and their relative proportion of sum of square (SSq G, SSq Treat and SSq GxTreat, respectively) are shown*.

*** P < 0.001;

* *P < 0.05; ns = non significant*.

Significant correlations were observed between traits in each treatment revealing the link between quality, phenology, and vegetative traits. To assess the repeatability of phenotyping measurement, single trait correlations between treatments within each experiment and among control treatments were evaluated. Most of the correlations were significant at *P* < 0.001 (Table 3). The strongest Pearson's correlation was found between FW and leaf in Exp.1, which exhibited a positive correlation. In Exp.2, the correlation between Flw and RIP was the strongest correlation. For both experiments, Flw and RIP were significantly and negatively correlated indicating that the later the truss flowered, the shorter the time to ripe. FW was also negatively correlated to SSC in every treatment. Across experiments, the sign of correlations were conserved for all significant correlations.

**Table 3:** Correlations among traits in each treatment and experiment.

**Ctrl1**							**Ctrl1-Ctr2**	**Ctrl2**							
Firm	Firm						−0.17	Firm	Firm						
Flw	**0.15**	Flw					**0.61**	Flw	ns	Flw					
FW	ns	**0.24**	FW				**0.4**	FW	ns	ns	FW				
Leaf	ns	**0.18**	**0.37**	Leaf			0.19	Leaf	ns	−0.15	ns	Leaf			
NFr	ns	ns	–**0.33**	ns	NFr		ns	NFr	ns	−0.15	–**0.24**	0.11	NFr		
RIP	**0.17**	–**0.26**	**0.22**	ns	–**0.28**	RIP	**0.49**	RIP	ns	–**0.28**	0.13	ns	–**0.18**	RIP	
SSC	−0.13	ns	–**0.17**	ns	ns	–**0.26**	**0.23**	SSC	ns	ns	–**0.25**	ns	ns	ns	
**WD**							**Ctrl1-WD**	**Salt**							**Ctrl2-SS**
Firm	Firm						**0.34**	Firm	Firm						0.19
Flw	ns	Flw					**0.86**	Flw	ns	Flw					**0.62**
FW	**0.18**	**0.14**	FW				**0.84**	FW	ns	–**0.29**	FW				**0.26**
Leaf	ns	**0.29**	**0.4**	Leaf			**0.34**	Leaf	ns	–**0.29**	0.15	Leaf			**0.46**
NFr	−0.11	ns	–**0.38**	−0.14	NFr		**0.55**	NFr	ns	–**0.19**	ns	ns	NFr		**0.31**
RIP	**0.2**	–**0.31**	**0.23**	ns	−0.12	RIP	**0.68**	RIP	−0.12	–**0.38**	ns	ns	ns	RIP	**0.5**
SSC	–**0.17**	ns	–**0.33**	–**0.17**	ns	–**0.31**	**0.6**	SSC	ns	**0.33**	–**0.22**	ns	ns	0.13	0.16

*Single trait correlation among controls (Ctrl1-Ctrl2) which is a measure of repeatability or between control and stress (Ctrl1-WD and Ctrl2-SS) is presented. Only significant correlations (P < 0.05) are indicated. They are in bold when significance is lower than 0.001. In bold P < 0.001; ns = non significant*.

### Impact of water deficit and salinity stress at phenotypic level

The effect of stress treatment was assessed by the mean relative variation (MV) calculated as detailed in Materials and Methods. In a first step, salinity and water deficit were compared to their relative control treatment in each experiment. In accordance to the results of ANOVA, FW, SSC, and Leaf were the traits most affected by stress treatments. Among all traits, SSC was the only one positively impacted by WD and SS with more than 10% increase compared to controls (Table 1). On average, when comparing each stress to its control, WD and SS affected all traits in the same direction except NFr. Indeed, NFr was reduced by WD condition (−15%) but slightly increased when comparing salinity to its control. FW and Leaf were both reduced in stress conditions while stress effects were less obvious for firmness and phenology traits.

For a convenient comparison of WD and SS applied in our study, we considered the control treatment in Exp.1 as reference, taking a subset of 241 lines commonly tested in all treatments. Indeed, the difference between the control in Exp.1 and treatments in Exp.2 lies mainly in the EC application that was 1.7 and 4.5 times higher in control of Exp.2 and SS, respectively. We then calculated the effect of those treatments compared to Ctrl1 and measured the effect of each of them in percentage of increase or decrease (Supplemental Figure 2). Using the same control revealed a growing negative effect of salt treatment while control in Exp.2 seemed to be intermediate between WD and SS.

**Figure 1:**
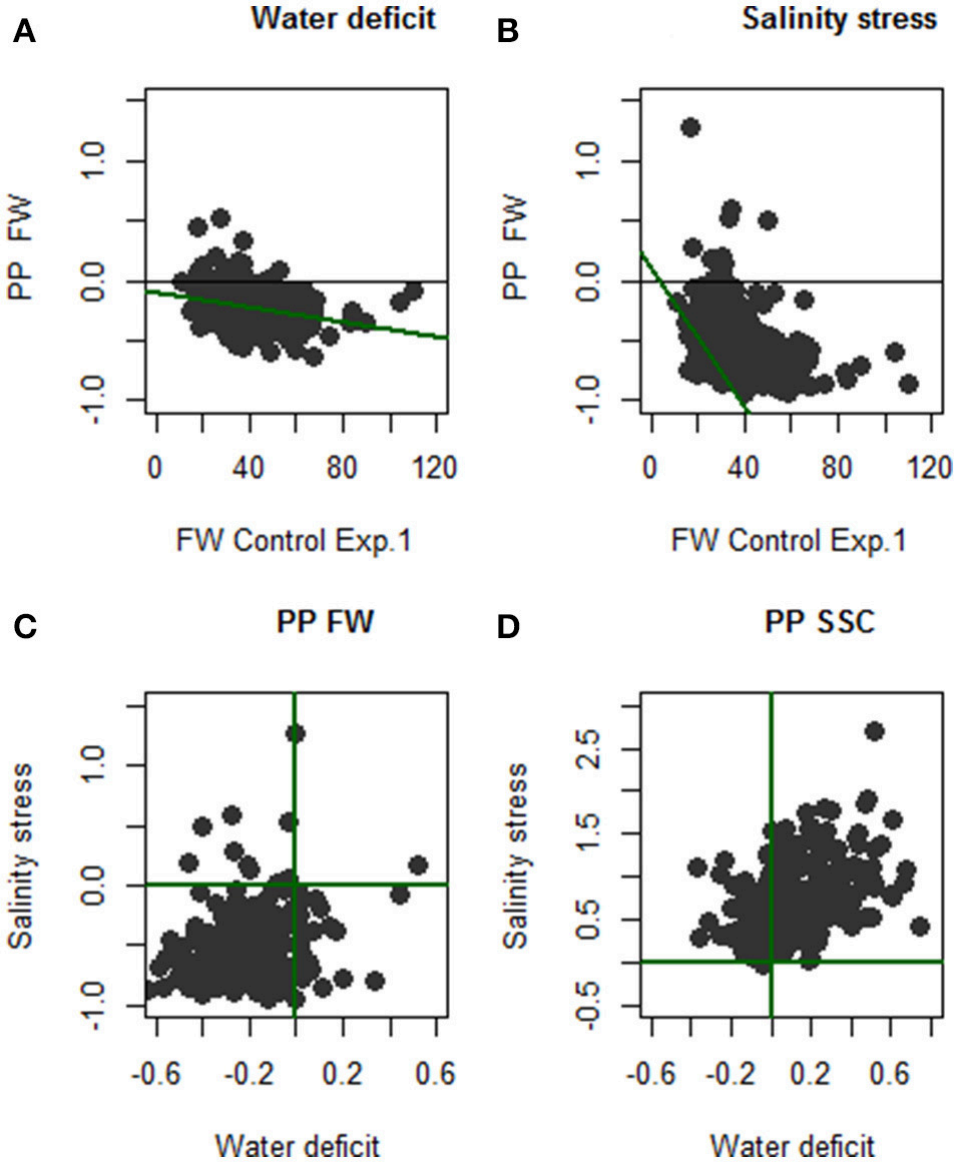
Effect of stress treatments on individual lines for FW and SSC. **(A,B)** plot the FW plasticity— which is the gain (above 0) or loss (below 0) of FW due to stress—against the FW in control treatment of Exp.1. **(C)** (respectively **D**) is the FW (respectively SSC) plasticity in WD against plasticity in SS treatment both compared to the same control in Exp.1.

Nevertheless, these average behaviors did not fully reflect the individual variations. FW plasticity was found negatively correlated to FW in control in both experiments, meaning that larger fruits were more affected by the stress. Indeed both stress decreased FW of accessions with fruits larger than 55g (Figures 1A,B). The plot of FW plasticity in SS against WD showed clearly that only one genotype had an increased FW in both conditions while 23 genotypes increased FW under WD and decreased it under SS and 10 genotypes react in the opposite direction (Figure 1C). For SSC, all genotypes except H10_84 increased SSC with SS treatment. Altogether, 67% of the genotypes increased SSC under both stresses pointing the possibility to improve sugar content in fruit by irrigation practices. However, as for FW, some genotypes were affected inconsistently by the stress treatments with 55 genotypes (22.8%) that increased SSC only in SS and not under WD (Figure 1D).

### QTL detection and stability

#### QTL detection

QTL mapping was performed using a genetic map constructed with 1,345 polymorphic SNP selected from the parental line resequencing data. This genetic map covers more than 84% of the genome and measures 2,156 cM (details in Pascual et al., 2015). With the available information of parental polymorphisms, the offspring haplotype structure was predicted by inferring the parental origin of each allele. On average, 88.7% of founder allele origin was accurately predicted with only 11% of the alleles that could not be strictly assigned to any parent (Supplemental Figure 3). Among the parents, Levovil and LA0147, with <10% of the allelic contribution in the MAGIC lines genome deviated, the most from the expected value of 12.5% of each parental allelic contribution.

**Figure 2:**
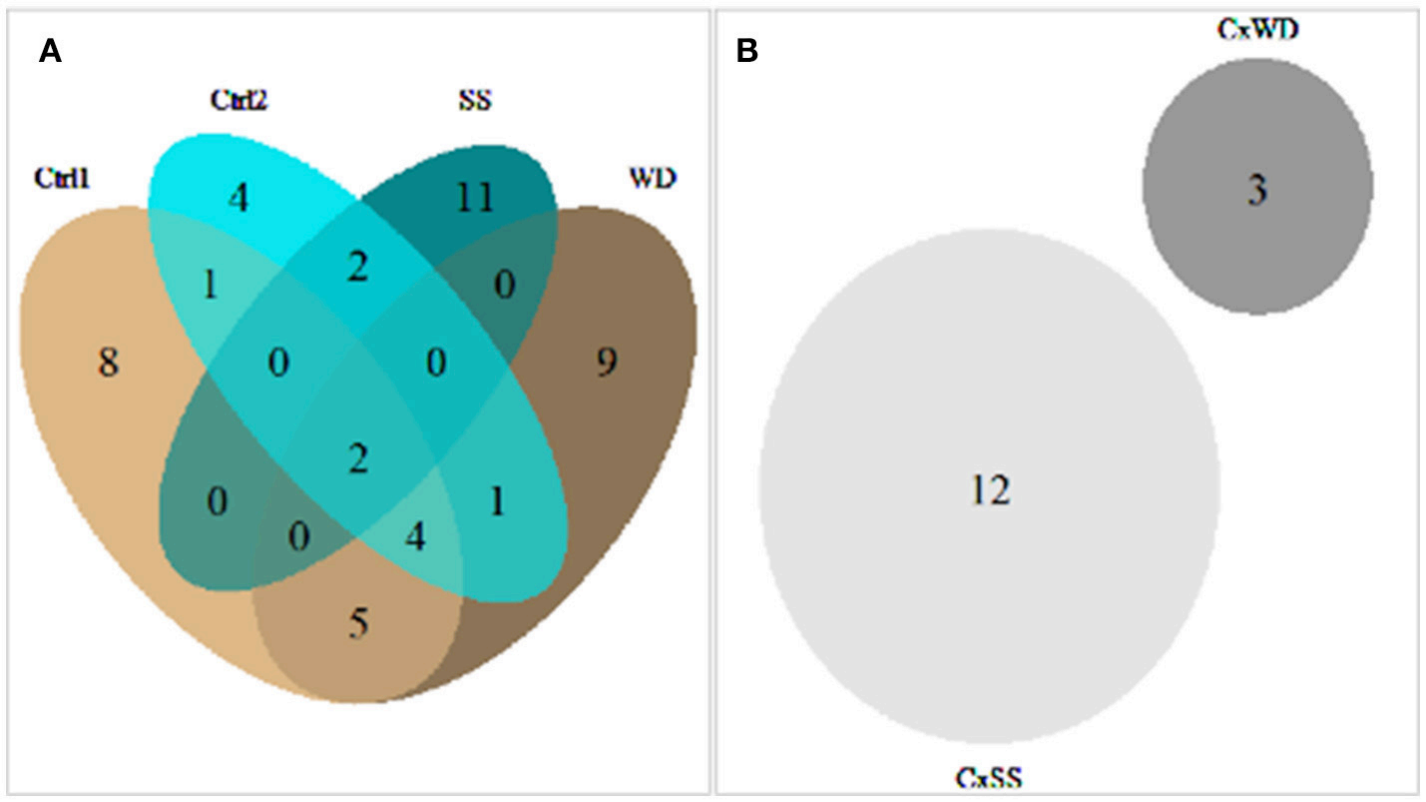
Venn diagram of the number of main effect QTL, detected on mean traits for all treatments **(A)** and interactive QTL, detected on plasticity traits for the two experiments **(B)**.

Considering all treatments, 54 QTL were identified for the seven traits evaluated and their plasticity. The number of QTL per trait varied from four for Flw to 11 for FW (Supplemental Table 1). Among these QTL, 19 were found in at least two treatments and around 65% (35 QTL) were treatment specific. Eleven QTL were common to WD and its control condition, while SS and its control condition shared only four QTL (Figure 2A).

Some QTL were specifically detected in one treatment or for plasticity traits (interactive QTL; Supplemental Table 1). Indeed, irrespective of interactive QTL, we observed eight and four QTL specific to control treatments for Exp.1 and Exp.2, respectively. Nine and 11 QTL were specific to WD and SS respectively, pointing that stress treatments present higher number of specific QTL. For interactive QTL, six were exclusively identified in Exp.2 and one in Exp.1 and no interactive QTL were shared between the two experiments (Figure 2B). This outlined the specificity of the interactive QTL. Confidence intervals (CI) of the QTL ranged from 4 to 60 cM (according to genetic distance) and 0.43 to 71.49 Mb (according to physical distance; Supplemental Table 1). The high number of recombination occurring in the MAGIC population allowed us to map 24 QTL with CI lower than 2Mb. The chromosome 11 presented the largest number of QTL, each trait except Flw presenting at least one QTL on this chromosome, whereas per trait, FW and SSC had the largest number of QTL (11 and 10, respectively).

#### Identification of interactive QTL (QTLxE)

We call interactive QTL (QTLxE) those mapped for plasticity traits in each experiment. Thus, for Exp.1, three QTLxE were detected for RIP (two QTL) and SSC. The RIP QTLs (*RIP9.1* and *RIP10.1*) were also mapped in control for Exp.1 and WD treatment, respectively. The QTLxE *SSC12.1* was specific to the interaction. Likewise, 12 QTLxE were mapped in Exp.2, among which six were specific to the interaction.

#### Co-localization of QTL

Clusters of QTL were localized especially on chromosomes 1, 2, 3, 10, and 11 (Supplemental Figure 4). Most of these QTL corresponded to correlated traits. For example, around 45 cM on chromosome 1, QTLs linked to phenology traits, FW, SSC, and NFr clustered and could be related to the pleiotropic effect of one QTL. The same observation was noted on chromosome 2 for quality traits and on chromosome 3 for phenology, quality and vegetative traits.

### Candidate gene selection

After the identification of constitutive and interactive QTLs, the number of genes and polymorphisms within the CI of any QTL mapped in a region lower than 2Mb was assessed using the sequencing information of all parental lines (Causse et al., 2013). For the 24 QTLs that had a CI shorter than 2 Mb, the number of genes within the CI (potential candidate genes) varied from 75 for *Leaf9.1* to 269 genes for *Firm11.1* with 3,804 and 12,530 polymorphisms associated, respectively (Table 4). We attempted to reduce the number of candidate genes (CG) by applying a filter in accordance to parental allelic effects at the QTL as described in Materials and Methods. This procedure was efficient for some QTL and allowed us to reduce the number of CG by nearly 80% of the total number of genes within the CI for *Firm11.1* and *Leaf10.1* Nevertheless, for *FW11.3 and RIP4.1* the parental allelic effect filtering wasn't efficient; none of the genes in the CI was discarded as a close haplotype was present in the region (Supplemental Figure 5).

**Table 4:** Characteristics of the 24 QTL with a confidence interval (CI) smaller than 2 Mb.

**QTL**	**CI Mb**	**Cervil**	**Levovil**	**Criollo**	**Stupicke**	**Plovdiv**	**LA1420**	**Ferum**	**LA0140**	**Nb.Genes**	**Nb.Pol**	**Filter**	**Nb.CG**	**Nb.CP**
*Firm1.1*	1.86	0.608	−1.266	−3.916	2.628	1.655	0.095	−0.629	0.824	245	10991	Criol # Stup	164	620
*Firm1.2*	1.05	−0.412	−0.539	−0.544	−0.541	−0.372	3.112	−0.428	−0.276	134	5630	LA14 # all	127	1719
*Firm11.1*	1.87	4.305	−0.651	−0.284	−2.169	−0.821	−0.435	−0.326	0.384	269	11903	Cerv # all	8	3
*Firm3.1*	1.44	−5.249	4.750	−3.842	3.354	−7.327	0.581	2.679	5.055	171	7051	Plov # (Lev = LA0)	36	29
*Firm8.1*	1.23	−0.411	−0.421	2.767	−0.815	−0.245	−0.636	−0.009	−0.231	117	7975	Criol # all	27	46
*Flw9.1*	1.00	0.292	1.523	−0.522	−1.460	3.004	−2.710	3.561	−3.690	119	6375	(Plov = Fer) # LA0	49	35
*FW11.2*	0.79	−0.131	0.664	−0.043	0.024	0.013	−0.040	0.003	−0.493	91	4478	Lev # LA0	29	32
*FW11.3*	1.42	−0.155	0.066	−0.049	0.130	−0.001	−0.022	*NA*	0.033	189	11532	Cerv # (Stup = Lev)	189	6989
*FW12.1*	0.43	0.008	−0.097	0.048	−0.142	−0.038	0.033	0.171	0.020	79	3407	Fer # (Stup = Lev)	77	562
*FW2.2*	1.74	−0.138	−0.031	0.050	−0.134	0.031	0.082	0.044	0.093	234	9957	(Cerv = Stup) # (LA14 = LA0)	52	1362
*FW3.2*	0.97	−0.049	−0.004	−0.087	0.056	0.162	−0.078	−0.005	0.003	122	6490	Criol # Plov	109	3026
*FW3.3*	1.52	−0.095	−0.007	−0.046	−0.038	0.056	−0.083	0.055	0.157	214	10182	(Cer = LA14) # LA0	142	377
*FW8.1*	1.63	−0.195	0.019	0.111	0.009	−0.132	0.046	0.097	0.047	180	7959	(Cer = Plov) # Criol	31	738
*Leaf10.1*	1.86	1.565	−0.622	2.628	−2.586	−0.070	3.058	−2.898	−1.073	264	13108	(Criol = LA14) # (Stup = Fer)	42	52
*Leaf11.1*	1.55	−2.416	2.005	2.040	3.103	−1.464	−2.799	0.890	−1.356	168	10084	Stup # (Cerv = LA14)	94	524
*Leaf3.1*	1.46	−2.985	2.944	−2.063	1.403	3.224	−1.286	−0.321	−0.918	193	9944	(Lev = Plov) # Cer	184	5803
*Leaf9.1*	0.76	0.798	−2.351	−1.744	5.031	−4.669	−2.079	3.154	1.861	75	3804	Plov # Stup	52	963
*NFr10.1*	1.53	−0.129	−0.021	0.039	0.354	0.063	−0.023	0.042	−0.326	212	5506	Stup # LA0	70	80
*RIP2.1*	1.74	−4.039	1.956	2.022	−3.948	−0.265	3.585	0.654	0.034	234	9957	(Cer = Stup) # LA14	103	1418
*RIP4.1*	1.21	−0.053	2.639	1.233	−0.053	1.788	−2.420	−0.653	−2.482	150	10794	(LA14 = LA0) # Lev	150	6629
*SSC1.2*	1.34	0.156	−0.250	−0.670	−0.207	0.194	−0.290	0.121	0.949	197	10528	LA0 # Criol	68	69
*SSC11.2*	1.56	0.970	−1.916	0.789	−0.594	0.018	0.398	*NA*	0.338	203	11813	(Cer = Criol) # Lev	78	681
*SSC12.1*	1.52	0.047	0.004	−0.007	−0.094	0.122	0.103	−0.153	−0.019	170	8232	(Plov = LA14) # Fer	110	395
*SSC4.1*	1.93	−0.792	0.363	0.214	0.441	0.811	0.252	−0.682	−0.606	211	15195	(Cerv = Ferum = LA0) # Plov	65	58

*The columns of the eight parents present their respective allelic effect for each QTL. Nb Genes and Nb.Pol count the number of genes and the number of polymorphisms identified—via the Solgenomic database—within the CI. After filtering these genes and polymorphisms according to the allelic effect of parents, the residual numbers of genes are counted as candidate genes (Nb.GC) with the residual number of candidate polymorphisms (N.CP). The parents chosen for the CG filtering are presented in the column “Filter” where the symbols = and # notified respectively parents where identical or divergent polymorphisms were kept*.

**Figure 3:**
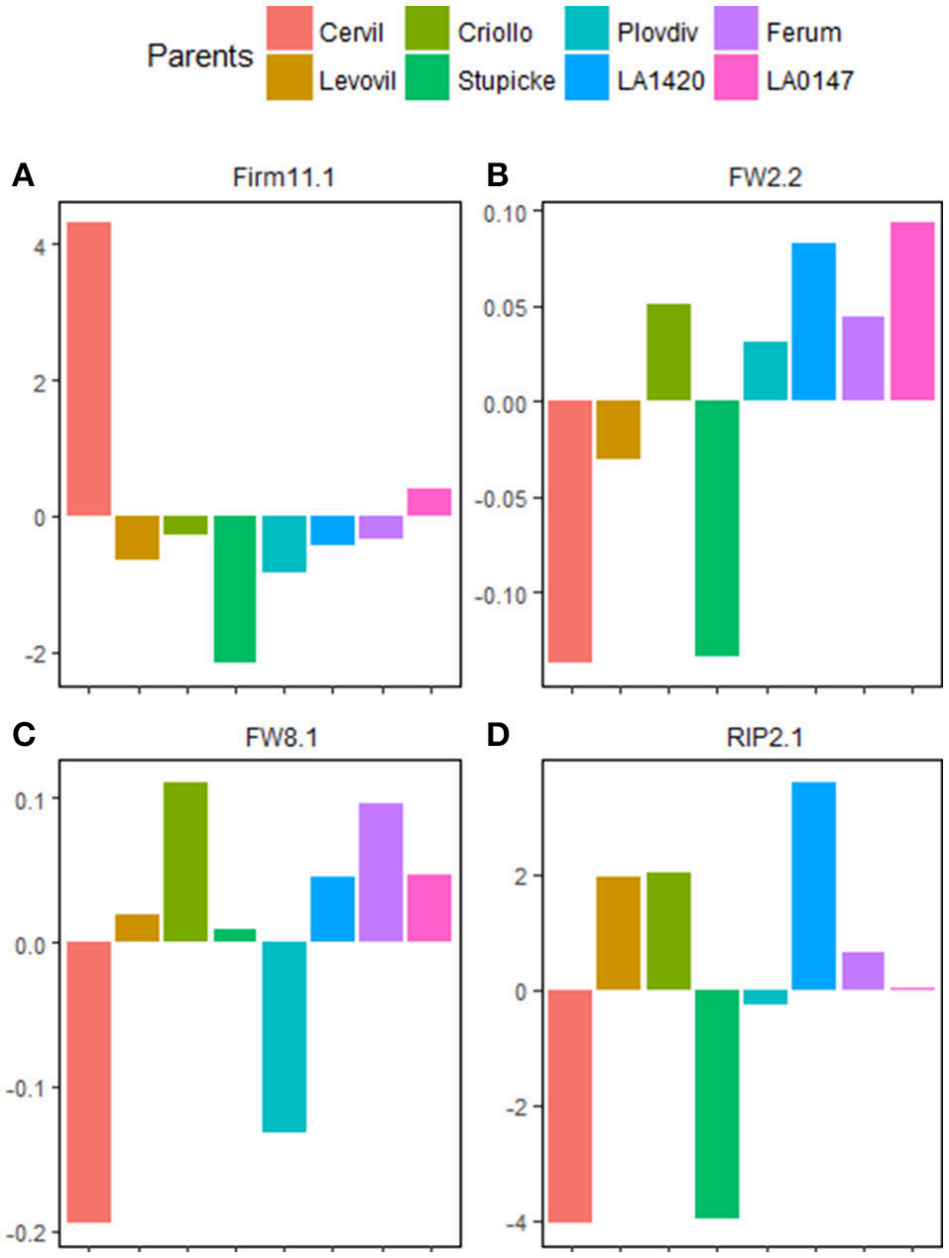
Example of QTL where the parental allelic effect allowed reducing the number of candidate genes. The QTL effects were mean centered to facilitate the visualization of allelic effect difference between parents. **(A)** Allelic effect of the eight parental lines for the QTL Firm11.1. **(B)** Allelic effect of the eight parental lines for the QTL FW2.2. **(C)** Allelic effect of the eight parental lines for the QTL FW8.1. **(D)** Allelic effect of the eight parental lines for the QTL RIP2.1.

The interactive QTL *Firm11.1*, identified in Exp.2 contained the largest number of genes within the CI (269 genes). Regarding the parental allelic effect at this QTL (Figure 3A), we filtered the candidates by keeping all polymorphisms that were specific to Cervil parent. This reduced the number of candidates to eight genes and polymorphisms with different effects (Supplemental Table 2). For *FW8.1*, we kept all polymorphisms identical between Cervil and Plovdiv and different from Criollo (Figure 3C), decreasing the number of CG to 31 genes (Supplemental Table 2). Five QTL presented <40 CG after the filtering procedure according to allelic parental effect variation (presented in Supplemental Table 2 with functional annotation of the CG). *FW2.2* QTL co-localized with a ripening time QTL *RIP2.1*. These two QTL shared the same CI comprising 1.74 Mb of length and containing 234 genes and 9,957 polymorphisms (Table 4). FW and RIP were highly positively correlated and could be impacted by one pleiotropic QTL. Moreover, *RIP2.1* and *FW2.2* presented the same pattern of parental allelic effect, at least for Cervil, Stupicke and LA1420 that had the strongest QTL effect (Figures 3B,D).

## Discussion

Parental lines of the MAGIC population did not include any wild accession (from the *S. pimpinellifolium* species) but had sufficient genetic diversity to allow QTL mapping on the offspring. The progeny exhibited a large variability with phenotypic transgressions in both directions in every tested condition (Supplemental Figure 1), suggesting new favorable allelic combinations obtained in the MAGIC population. Besides, the slight impact of WD and SS on the heritability suggests possibility for marker-assisted selection (MAS). Huang et al. (2015) proposed an interesting MAS approach for MAGIC populations called Multi-parent advanced generation recurrent selection (MAGReS) involving the inter-cross of individuals with the best allelic combinations for one (or more) trait(s) of interest to produce highly performant RILs. The MAGIC population tested here is thus a valuable resource to apply such breeding strategy. However, our results showed high level of GxE for the two experiments that affect also the QTL detection, as 35 QTL (65%) were specifically detected on one condition. Furthermore, FW and SSC, the most important agronomic traits, carried ten or more QTL in all condition tested with only one QTL (*FW2.2*) stable across all treatments. For these traits, MAS may not be of great utility for breeding programs targeting variable cultural areas. Thus, the breeding strategy should take into account the specificity of the QTL to achieve optimal benefit per environment. Applying the MAGReS strategy by selecting genotypes to inter-cross following the performance per environment in order to achieve rapidly performing crop, is an innovative approach to sustain breeding effort.

On average, WD and SS impacted sugar content, fruit weight and leaf length more than the other traits. They both reduced FW and Leaf while SSC was the only trait positively affected by up to 10% increase with respect to control in Exp.1 (Supplemental Figure 2). Similar results were frequently found in the literature (Villalta et al., 2007; Huang et al., 2009). The higher SSC under WD and SS was assumed to derive from the fruit water content reduction without necessarily involving higher synthesis of soluble sugar. Indeed, several studies reveal a negative correlation between FW and SSC, pointing a physiological link of these two traits making a simultaneous improvement difficult to be achieved. However, Navarro et al. (2005) showed that when SS occurs, the increased concentrations of sugars and acids were probably both due to the decrease in water content in the fruit and additionally to new sugars synthesis, since concentrations calculated on a dry weight basis also increased. Our results showed 20 and 11 genotypes that increased simultaneously FW and SSC under WD and SS respectively. This may be linked to a positive regulation of SSC during drought and salinity. These genotypes are interesting for quality improvement in tomato with minor impact on FW.

The results of the QTL analyses confirmed the polygenic architecture of fruit quality traits. SSC and FW that are among the most important fruit quality traits had the highest number of QTL identified. Besides this polygenic architecture, the positions of these QTL are distributed along the genome. QTLs related to FW and SSC were identified on six and seven chromosomes respectively, considering all treatments but treatment specific QTL were also identified. In optimal growth condition (Control of Exp.1), seven FW QTL (out of the 11 QTL mapped for FW) were identified, explaining additionally 68.68% of the phenotypic variation, while only one SSC QTL (*SSC2.1*; out of the 10 SSC QTL) was identified, with 6.98% of phenotypic variation. This suggests that SSC QTLs are easier detected in stress than control conditions.

Among all the QTL identified in this study, 35 QTL were treatment specific and only two QTL (*Flw1.1* and *FW2.2*) were stable across every treatment. Depending on the environmental conditions, the main QTL responsible of the observed phenotypic variation are not the same. Only one third of the QTL were detected in at least two treatments. These results reinforce the idea of targeted environment breeding strategy in order to achieve better results per environment.

Fifteen interactive QTL were identified, three in Exp.1 and 12 in Exp.2 but none of them co-localized between the two experiments suggesting different genetic control of the phenotypic plasticity under WD and SS. Two main ideas were developed concerning the genetic control of phenotypic plasticity advocating that: (i) phenotypic plasticity can be caused by environmentally sensitive loci associated with a phenotype, directly influencing the trait value in both environments; (ii) or it can be caused by regulatory genes that simply influence the plasticity of a phenotype. This means that plasticity can be viewed as the result of the action of alleles that have different effects in different environments or being under the control of regulatory loci (Via et al., 1995). Besides, QTL mapping study can be used to address easily which one of these hypotheses is the most probable (Ungerer et al., 2003; Tétard-Jones et al., 2011). When plasticity QTL co-localized with QTL mapped on mean trait value in at least one of the environment tested, they are assumed to be under the control of allelic sensitivity loci. On the contrary, QTL that are specific to plasticity are mainly linked to regulatory genes. In Exp.1 three QTLxE were identified: *RIP9.1, RIP10.1* and *SSC1.2* but only the last one was specific to the interaction. At the same time in Exp.2, among the 12 QTLxE, six were specific to the interaction. One can assume these QTLxE to be under regulation of WD (Exp.1) and SS (Exp.2) response genes, which make them particularly interesting for breeding in stressful environment.

Multi-parental populations offer new insight into fine mapping of quantitative traits (Kover et al., 2009; Milner et al., 2016). The high recombination events occurring in this type of population in addition to the infinite possibility of repeated study are of major interest. One advantage is the high allele segregation compared to bi-parental population and low LD with poor structure compared to GWAS, making them intermediate and complementary between these types of mapping populations (Pascual et al., 2016). Our results were compared to those of Albert et al. (2016a) and Albert et al. (2016b), that were conducted respectively on bi-parental population and a GWAS panel of tomato grown in similar condition of control and WD treatment than Exp.1. Among the 30 QTL identified in Exp.1, 18 QTL (60%) were also detected in the GWAS or RILs population, but only *Firm11.1* and *SSC11.2* were shared between the three panels. The ability to map QTL considerably depends in the mapping population pointing the relevance of combining different mapping population to identify stable QTL and balanced the advantage and disadvantage of each type of population.

The parental allelic information in the MAGIC population is a real advantage to screen and reduce candidate polymorphisms within the CI of a QTL as first described in Pascual et al., (2015). Indeed, the parents of the MAGIC population present very diverse allelic effects depending on the QTL. Some QTL had very divergent parental allelic effect while some other showed one parent varying differently from others. For example, *Firm1.2, Firm8.1*, and *Firm11.1* had all one parent divergent that seem to carry the allele responsible of the phenotypic variation. Besides, those QTL present very strong percentages of variation explained, that makes them interesting targets for breeding. These effects efficiently facilitate the filtering procedure to reduce CG.

On the chromosome 2, in a nearly 8 Mb region ranging from 44.55 to 52.92 Mb, two FW QTL were identified in our study. However, this region contains at least three already known QTL impacting fruit size and fruit shape, two of them positionally cloned: the fruit weight 2.2 (Solyc02g090740) cloned by Frary et al. (2000) and the ovate locus (Solyc02g085500) cloned by Liu et al. (2002). A third FW QTL was fine mapped by Muños et al. (2011) in this region, corresponding to a locule number (lc) locus. The first QTL identified on the chromosome 2 in our study (*FW2.1*) falls in a region of 3.5 Mb covering the *lc* and *ovate* loci. 462 genes and 20,742 polymorphisms were present in this region, and the filtering procedure did not efficiently reduce the CG. The second FW QTL on chromosome 2 (*FW2.2*) felt in a region of 1.74 Mb and covered the QTL fw2.2 cloned by Frary et al. (2000). However, this QTL was discarded when we attempt to reduce CG according to allelic effect of Cervil and Stupicke (Figure 3B). This suggests a second FW QTL closely linked to fw2.2. Nevertheless, Pascual et al. (2015) suggested a possible bias in the estimation of allelic parent's effect in regions where many QTL for a given trait are present. Indeed, in this case, the bias of allelic effect estimation may arise if different allelic combinations control different QTL. The QTL were mapped by interval mapping procedure meaning that each interval was tested for linkage with the phenotype. A whole genome mapping method, as proposed by Verbyla et al. (2014) for MAGIC populations, would better capture all small effect QTL and may limit the bias in QTL effect estimation. Anyhow the region of *FW2.2* is of great interest since several studies conducted on different mapping populations identified FW QTL within (Pascual et al., 2015; Albert et al., 2016a).

The number of candidate genes and polymorphisms was reduced using the parental re-sequencing information that allowed comparison of parental genotypes within CI of any detected QTL. Five QTL presented <40 CG after the filtering procedure (Supplemental Table 2). For these QTL, the putative functions of CG were screened according to the tomato genome annotation (SL2.50). Eight CG were retained for the QTL *Firm11.1* and all the polymorphisms related to these CG were on intergenic regions (Modifier effect in Supplemental Table 2). Among these CG, only Solyc11g006210 was not annotated. The functional annotation of the seven others highlighted one interesting CG (Solyc11g005820) which is a pectinesterase inhibitor. Pectinesterase inhibitors are involved in the rigidification or loosening of the cell wall. Thus, the Solyc11g005820 gene constitutes a good candidate for firmness variation.

*FW8.1* presented 31 CG after the filtering procedure but the number of candidate polymorphisms was very high (Supplemental Table 2). In this region, most of the CG were affected by more than one polymorphism pointing the need of deeper characterization of our candidate regions to confirm the effectiveness of causal polymorphisms. Nevertheless, this region carried three SNP that had a high effect modifying splice site or start/stop codon whereas most of the candidate polymorphisms remaining after the allelic filtering for other QTL were located in intergenic regions. The three SNP with high effect in the CI of *FW8.1* affected the genes Solyc08g075430, Solyc08g075470, and Solyc08g075510.

We showed in this study the presence of high level of genotype × environment interaction and how these interactions affect the QTL detection according to the environment. Specific and constitutive QTL were identified—in high precision for some—for phenology, vegetative and quality traits and the availability of the parental sequence information was useful for the genetic and genomic characterization of polymorphisms responsible for trait variation. The parental sequences allowed filtering CG and polymorphisms for the QTL mapped on regions carrying divergent parental haplotypes. The transcriptomic response through RNA-sequencing analyses on all parental lines should offer additional information that will be used to improve and support the CG selection. Functional validation could be envisaged afterward in order to detect the exact causal polymorphisms under the QTL of interest.

## Author contributions

This work is a part of the Ph.D. project of ID who conducted the statistical analyses and the redaction of the manuscript. LD conducted the experimental trials and phenotyping in Morocco. FB developed the bioinformatics tools to identify the polymorphisms on the MAGIC parental lines. LP reviewed the manuscript and shared a part of the script for QTL mapping analysis. MC supervised all the process of this work, constructed the experimental design and monitored the redaction of the article.

### Conflict of interest statement

The authors declare that the research was conducted in the absence of any commercial or financial relationships that could be construed as a potential conflict of interest.

## Supplementary material

Supplemental Figure 1 Distribution of mean values across MAGIC lines for each trait in Exp.1 **(A)** and Exp.2 **(B)**; For each trait, minimum (dotted lines) and maximum (solid lines) parental values are plotted for control (green) and stress (red) treatment.

Supplemental Figure 2 Average variation caused by water deficit (WD), salinity (SS) and control in Exp.2 (Ctrl2) relative to control in Exp.1. The effect of each treatment was measured in percentage of increase or decrease against control in Exp.1.

Supplemental Figure 3 Haplotype prediction. Each of the 12 tomato chromosomes is represented with the percentage of allelic contribution of every parental line. NA represented all positions on the chromosomes where the parental allelic origin could not be assigned.

Supplemental Figure 4 Mapchart representation of detected QTL on the genetic map for all chromosomes where a QTL was identified. The dashes on the chromosomes barchart represent the centimorgan distances between markers along the chromosomes. Each trait has a color code representation.

Supplemental Figure 5 Allelic effect of parental lines for QTL that were mapped in a confidence interval smaller than 2Mb.

Supplemental Table 1 QTL detected in the different conditions. For each trait, all the QTL found are identified by a specific name (QTL name column), the treatment where the QTL was found (Treatment), the chromosome (Chr) and the position (Pos) in cM. The peak region encompassing any QTL is defined by a pair of marker (LeftMrk and RightMrk), corresponding to the lower and upper bound expressed in genetic distances (Lower cM & Upper cM) as well as physical distances (Lower Mb & Upper Mb). The confidence interval in centimorgan (CI cM) and Mega-base (CI Mb) represent the corresponding differences between Upper and Lower. R2 is the percentage of phenotypic variation explained by a QTL. Ctrl 1 and Ctrl2 are the controls treatment for Exp.1 and Exp.2 respectively where the QTL were found. WD and SS are the stress treatment for water deficit and salinity. When interactives QTL are identified in Exp.1 (respectively Exp.2) treatment CxWD (respectively (CxSS) are the corresponding treatment designed.

Supplemental Table 2 Functional annotation of CG retained after the filtering procedure according to allelic parental effect. Only QTL that presented <40 CG were screened. For each QTL, the chromosome and localization (position in pb) were precised. The type of polymorphisms, depending if it is a single nucleotide polymorphism (snp) or insertion deletion (indel) were in the column “Type.” The number of snp or indel at a given gene is marked in brackets () when more than one polymorphism affected a given gene. The impact of polymorphisms affecting a gene were defined as MODIFIER when snp or indel are located in upstream or downstream region; MODERATE and LOW when polymorphisms had respectively non-synonymous or synonymous variant effect and HIGH when they affect splice site variant or start/stop codon. The putative function of each CG was checked on the annotation database of the tomato genome assembly (SL2.50).
