# Supplementary figures and images for: Water Deficit and Salinity Stress Reveal Many Specific QTL for Plant Growth and Fruit Quality Traits in Tomato
Source: Front Plant Sci. 2018 Mar 6;9:279. doi: 10.3389/fpls.2018.00279 (PMC5845638; doi:10.3389/fpls.2018.00279)

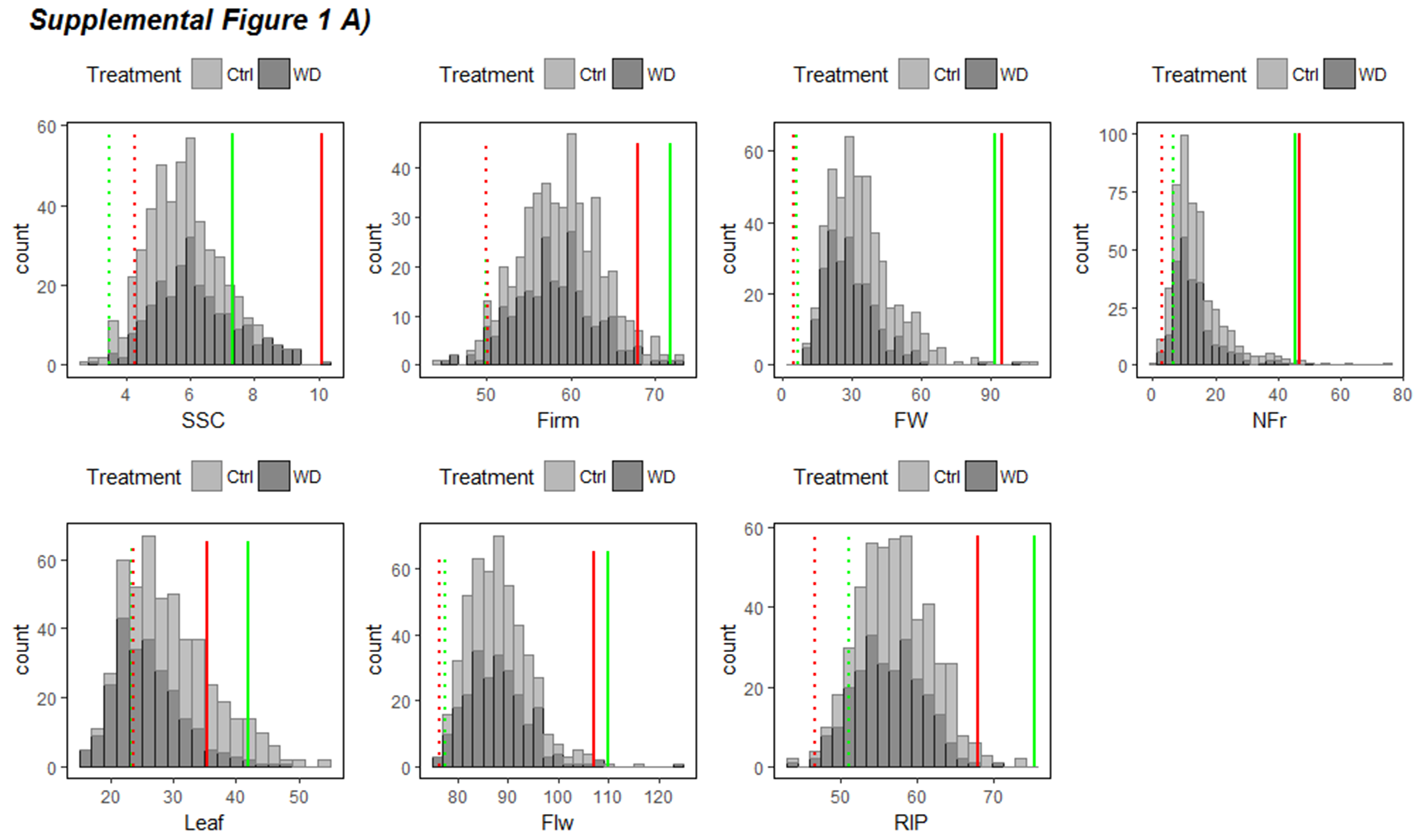

Supplement: Supplemental Figure 1 — Distribution of mean values across MAGIC lines for each trait in Exp.1 (A) and Exp.2 (B); For each trait, minimum (dotted lines) and maximum (solid lines) parental values are plotted for control (green) and stress (red) treatment. [file Image1.tiff]

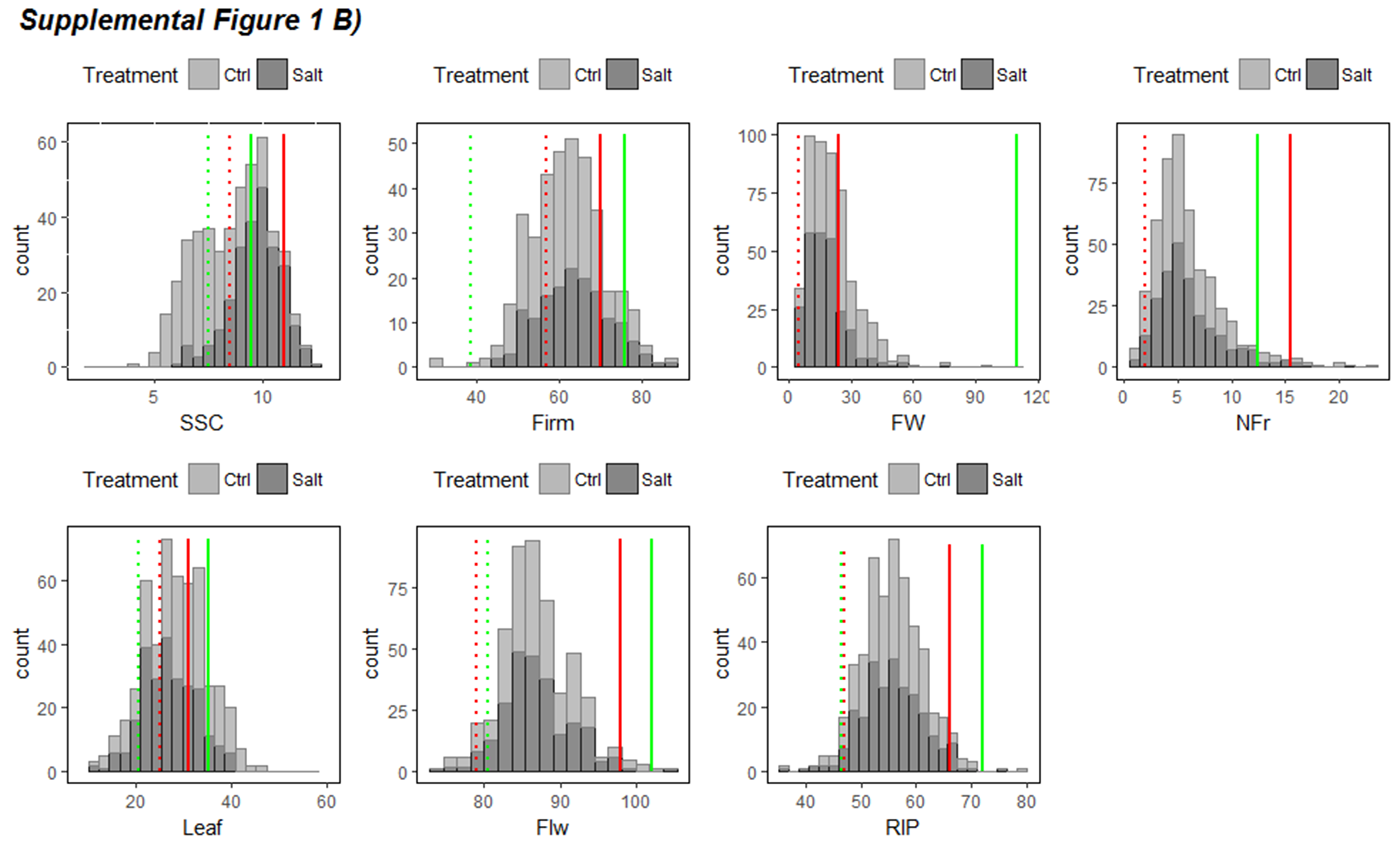

Supplement: Supplemental Figure 2 — Average variation caused by water deficit (WD), salinity (SS) and control in Exp.2 (Ctrl2) relative to control in Exp.1. The effect of each treatment was measured in percentage of increase or decrease against control in Exp.1. [file Image2.tiff]

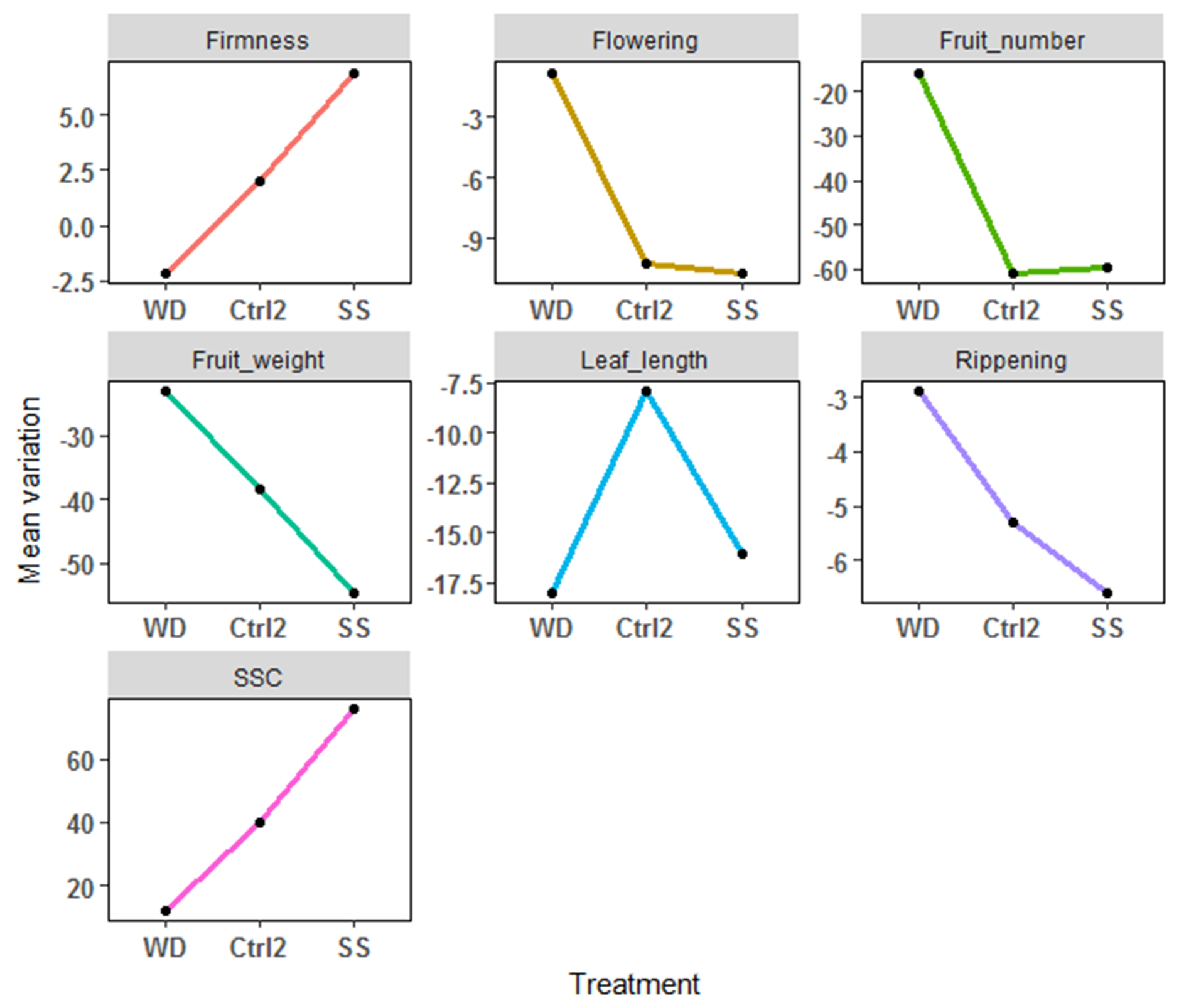

Supplement: Supplemental Figure 3 — Haplotype prediction. Each of the 12 tomato chromosomes is represented with the percentage of allelic contribution of every parental line. NA represented all positions on the chromosomes where the parental allelic origin could not be assigned. [file Image3.TIFF]

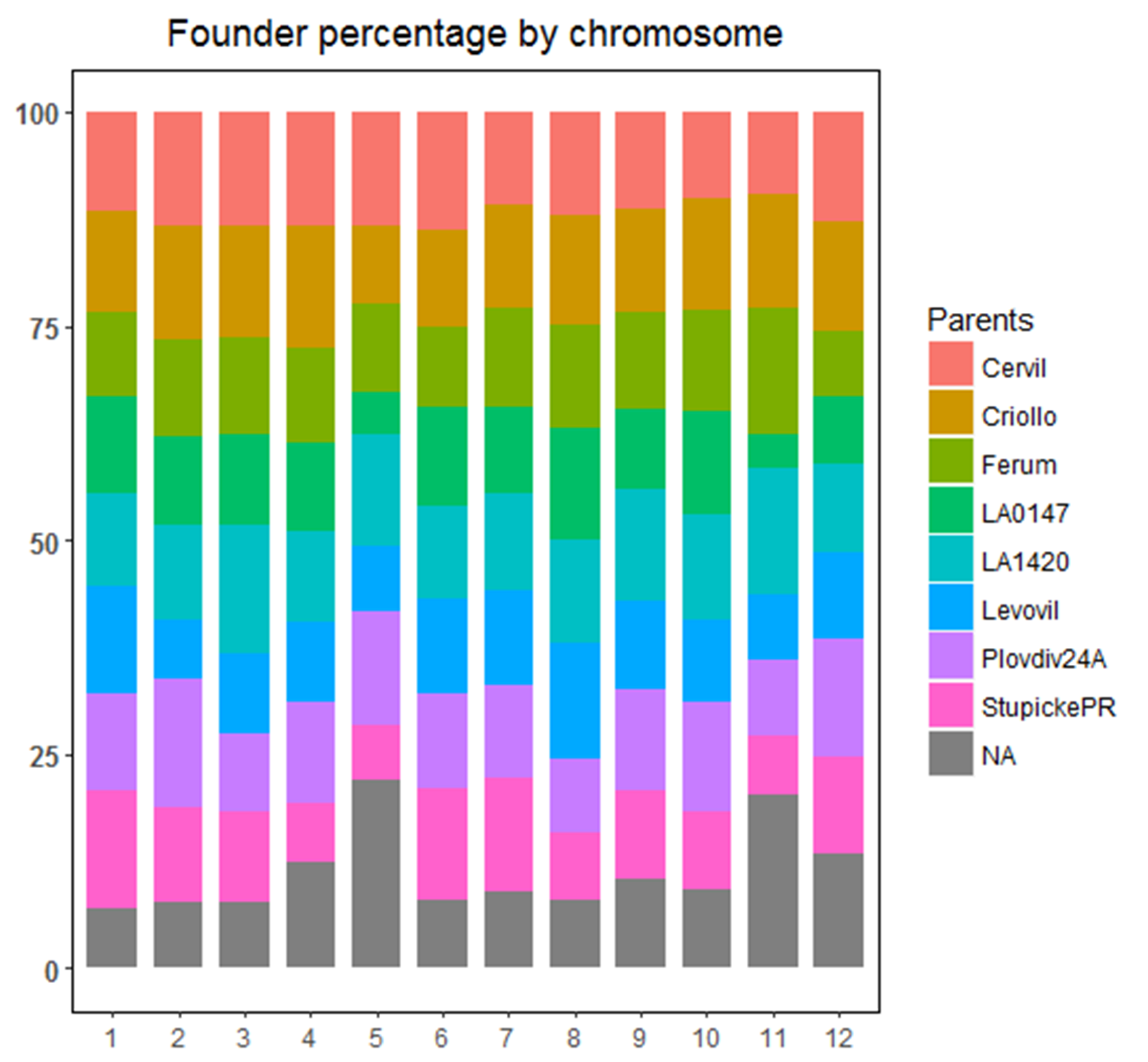

Supplement: Supplemental Figure 4 — Mapchart representation of detected QTL on the genetic map for all chromosomes where a QTL was identified. The dashes on the chromosomes barchart represent the centimorgan distances between markers along the chromosomes. Each trait has a color code representation. [file Image4.TIFF]

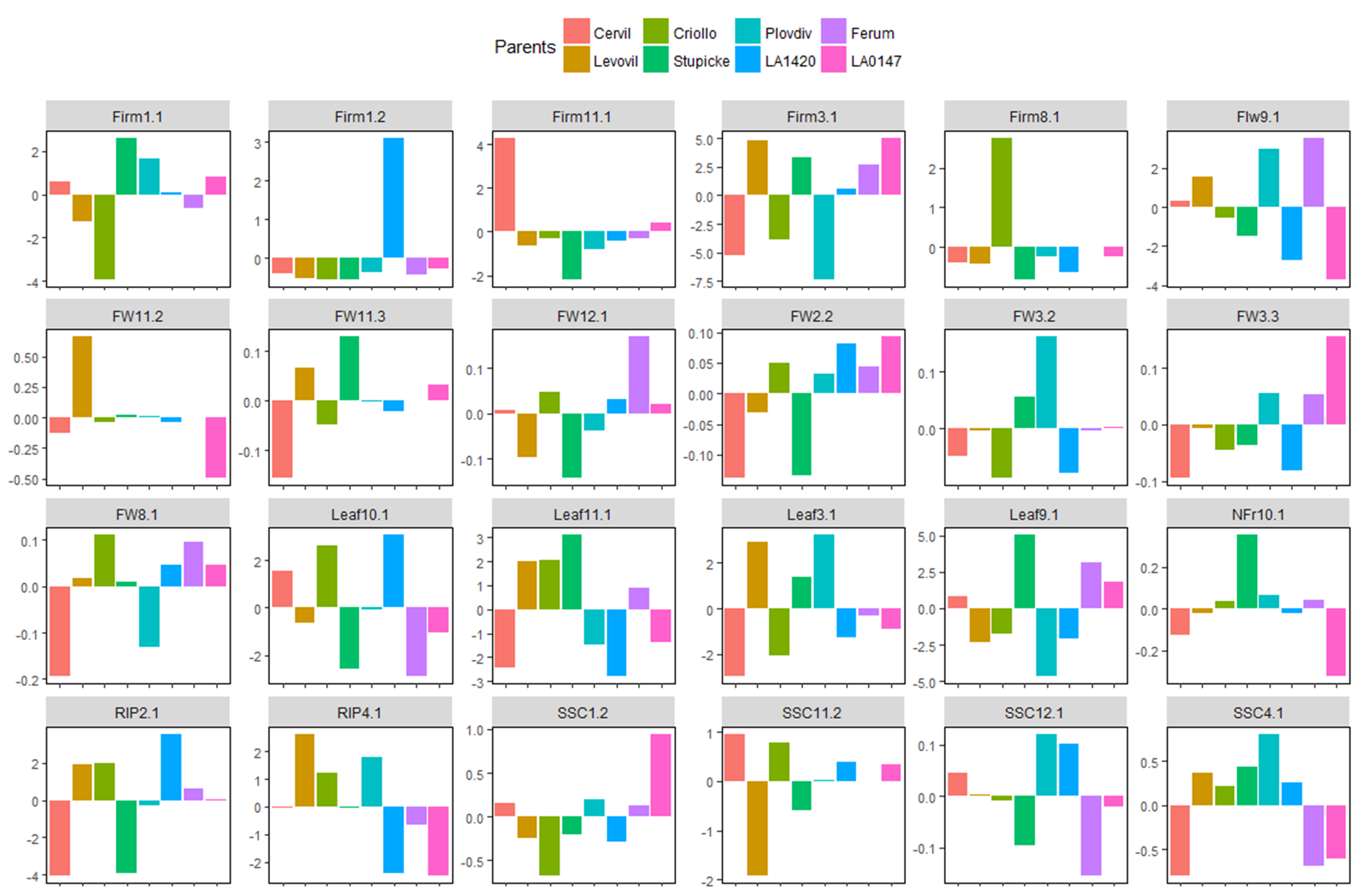

Supplement: Supplemental Figure 5 — Allelic effect of parental lines for QTL that were mapped in a confidence interval smaller than 2Mb. [file Image5.TIFF]
