# Supplementary material for: Water Deficit and Salinity Stress Reveal Many Specific QTL for Plant Growth and Fruit Quality Traits in Tomato
Source: Front Plant Sci. 2018 Mar 6;9:279. doi: 10.3389/fpls.2018.00279 (PMC5845638; doi:10.3389/fpls.2018.00279)
Supplement: Supplementary file 7 [file DataSheet1.PDF]

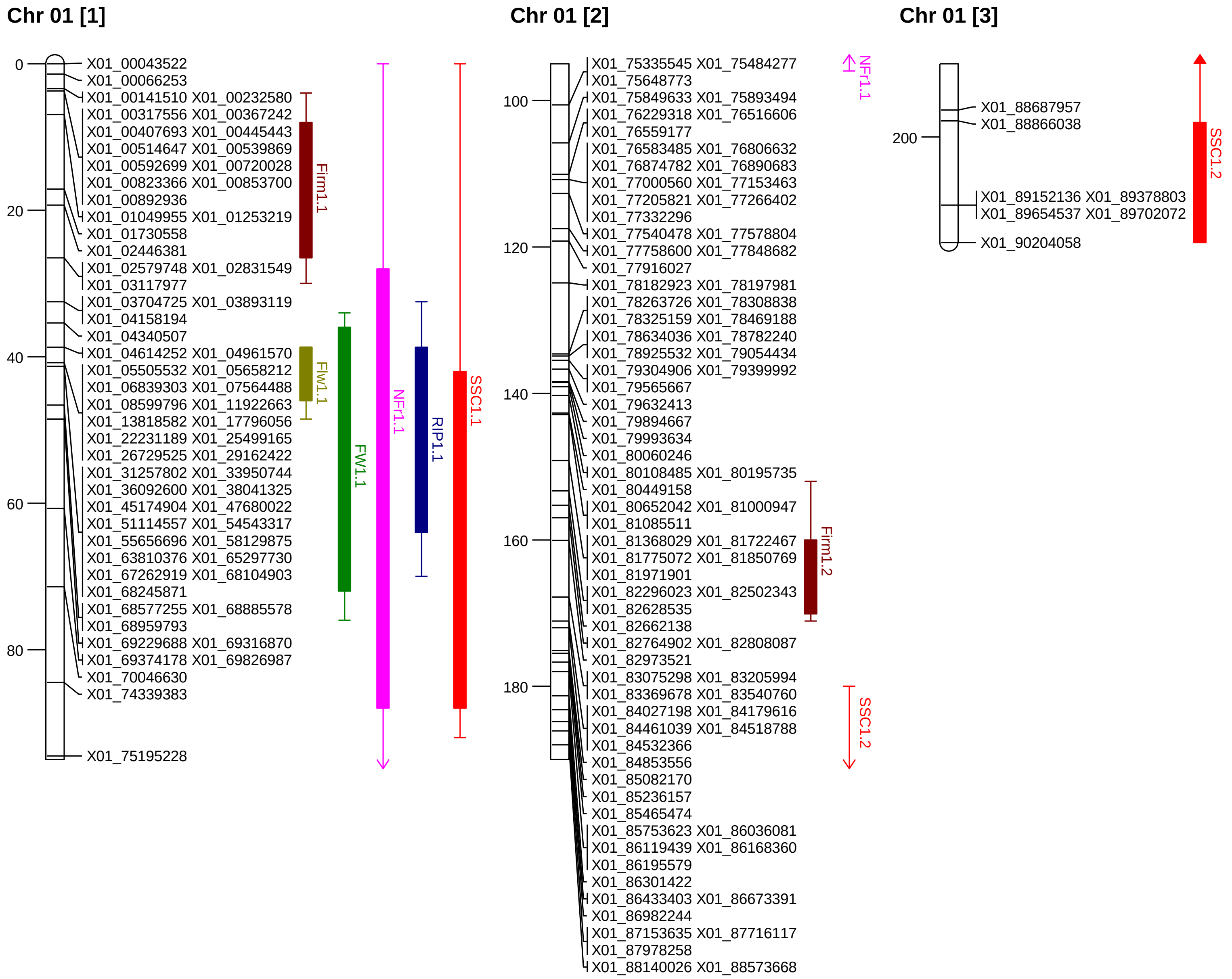
Chr 01 [1]
Chr 01 [2]
Chr 01 [3]
0
20
40
60
80
100
120
140
160
180
200
X01_00043522
X01_00066253
X01_00141510 X01_00232580
X01_00317556 X01_00367242
X01_00407693 X01_00445443
X01_00514647 X01_00539869
X01_00592699 X01_00720028
X01_00823366 X01_00853700
X01_00892936
X01_01049955 X01_01253219
X01_01730558
X01_02446381
X01_02579748 X01_02831549
X01_03117977
X01_03704725 X01_03893119
X01_04158194
X01_04340507
X01_04614252 X01_04961570
X01_05505532 X01_05658212
X01_06839303 X01_07564488
X01_08599796 X01_11922663
X01_13818582 X01_17796056
X01_22231189 X01_25499165
X01_26729525 X01_29162422
X01_31257802 X01_33950744
X01_36092600 X01_38041325
X01_45174904 X01_47680022
X01_51114557 X01_54543317
X01_55656696 X01_58129875
X01_63810376 X01_65297730
X01_67262919 X01_68104903
X01_68245871
X01_68577255 X01_68885578
X01_68959793
X01_69229688 X01_69316870
X01_69374178 X01_69826987
X01_70046630
X01_74339383
X01_75195228
Firm1.1
Flw1.1
FW1.1
NFr1.1
RIP1.1
SSC1.1
X01_75335545 X01_75484277
X01_75648773
X01_75849633 X01_75893494
X01_76229318 X01_76516606
X01_76559177
X01_76583485 X01_76806632
X01_76874782 X01_76890683
X01_77000560 X01_77153463
X01_77205821 X01_77266402
X01_77332296
X01_77540478 X01_77578804
X01_77758600 X01_77848682
X01_77916027
X01_78182923 X01_78197981
X01_78263726 X01_78308838
X01_78325159 X01_78469188
X01_78634036 X01_78782240
X01_78925532 X01_79054434
X01_79304906 X01_79399992
X01_79565667
X01_79632413
X01_79894667
X01_79993634
X01_80060246
X01_80108485 X01_80195735
X01_80449158
X01_80652042 X01_81000947
X01_81085511
X01_81368029 X01_81722467
X01_81775072 X01_81850769
X01_81971901
X01_82296023 X01_82502343
X01_82628535
X01_82662138
X01_82764902 X01_82808087
X01_82973521
X01_83075298 X01_83205994
X01_83369678 X01_83540760
X01_84027198 X01_84179616
X01_84461039 X01_84518788
X01_84532366
X01_84853556
X01_85082170
X01_85236157
X01_85465474
X01_85753623 X01_86036081
X01_86119439 X01_86168360
X01_86195579
X01_86301422
X01_86433403 X01_86673391
X01_86982244
X01_87153635 X01_87716117
X01_87978258
X01_88140026 X01_88573668
NFr1.1
Firm1.2
SSC1.2
X01_88687957
X01_88866038
X01_89152136 X01_89378803
X01_89654537 X01_89702072
X01_90204058
SSC1.2

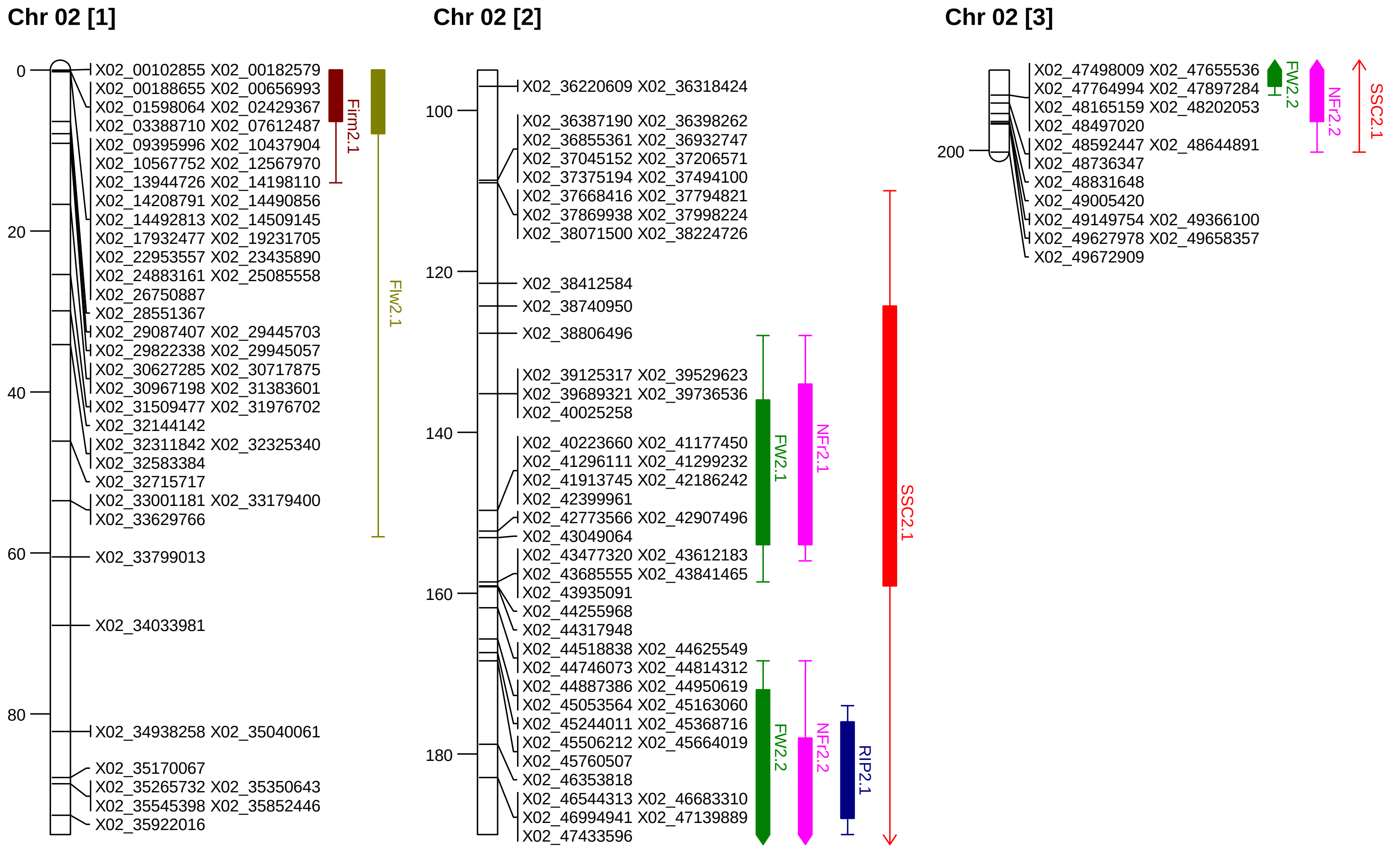
Chr 02 [1]
Chr 02 [2]
Chr 02 [3]
X02_00102855 X02_00182579
X02_00188655 X02_00656993
X02_01598064 X02_02429367
X02_03388710 X02_07612487
X02_09395996 X02_10437904
X02_10567752 X02_12567970
X02_13944726 X02_14198110
X02_14208791 X02_14490856
X02_14492813 X02_14509145
X02_17932477 X02_19231705
X02_22953557 X02_23435890
X02_24883161 X02_25085558
X02_26750887
X02_28551367
X02_29087407 X02_29445703
X02_29822338 X02_29945057
X02_30627285 X02_30717875
X02_30967198 X02_31383601
X02_31509477 X02_31976702
X02_32144142
X02_32311842 X02_32325340
X02_32583384
X02_32715717
X02_33001181 X02_33179400
X02_33629766
X02_33799013
X02_34033981
X02_34938258 X02_35040061
X02_35170067
X02_35265732 X02_35350643
X02_35545398 X02_35852446
X02_35922016
Firm2.1
Flw2.1
X02_36220609 X02_36318424
X02_36387190 X02_36398262
X02_36855361 X02_36932747
X02_37045152 X02_37206571
X02_37375194 X02_37494100
X02_37668416 X02_37794821
X02_37869938 X02_37998224
X02_38071500 X02_38224726
X02_38412584
X02_38740950
X02_38806496
X02_39125317 X02_39529623
X02_39689321 X02_39736536
X02_40025258
X02_40223660 X02_41177450
X02_41296111 X02_41299232
X02_41913745 X02_42186242
X02_42399961
X02_42773566 X02_42907496
X02_43049064
X02_43477320 X02_43612183
X02_43685555 X02_43841465
X02_43935091
X02_44255968
X02_44317948
X02_44518838 X02_44625549
X02_44746073 X02_44814312
X02_44887386 X02_44950619
X02_45053564 X02_45163060
X02_45244011 X02_45368716
X02_45506212 X02_45664019
X02_45760507
X02_46353818
X02_46544313 X02_46683310
X02_46994941 X02_47139889
X02_47433596
FW2.1
NFr2.1
SSC2.1
FW2.2
NFr2.2
RIP2.1
X02_47498009 X02_47655536
X02_47764994 X02_47897284
X02_48165159 X02_48202053
X02_48497020
X02_48592447 X02_48644891
X02_48736347
X02_48831648
X02_49005420
X02_49149754 X02_49366100
X02_49627978 X02_49658357
X02_49672909
FW2.2
NFr2.2
SSC2.1
0
20
40
60
80
100
120
140
160
180
200

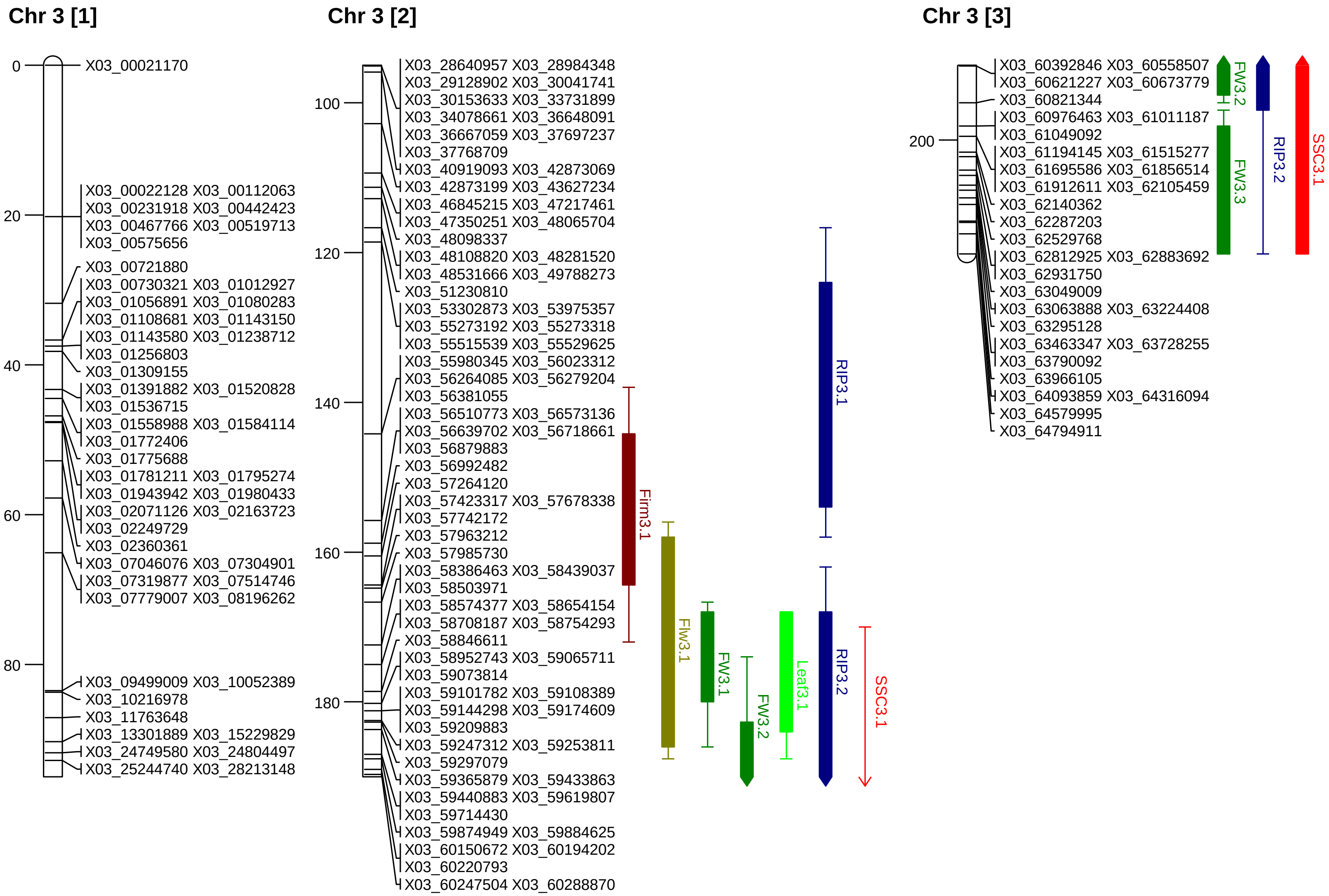
Chr 3 [1]
0
20
40
60
80
X03_00021170
X03_00022128 X03_00112063
X03_00231918 X03_00442423
X03_00467766 X03_00519713
X03_00575656
X03_00721880
X03_00730321 X03_01012927
X03_01056891 X03_01080283
X03_01108681 X03_01143150
X03_01143580 X03_01238712
X03_01256803
X03_01309155
X03_01391882 X03_01520828
X03_01536715
X03_01558988 X03_01584114
X03_01772406
X03_01775688
X03_01781211 X03_01795274
X03_01943942 X03_01980433
X03_02071126 X03_02163723
X03_02249729
X03_02360361
X03_07046076 X03_07304901
X03_07319877 X03_07514746
X03_07779007 X03_08196262
X03_09499009 X03_10052389
X03_10216978
X03_11763648
X03_13301889 X03_15229829
X03_24749580 X03_24804497
X03_25244740 X03_28213148
Chr 3 [2]
100
120
140
160
180
X03_28640957 X03_28984348
X03_29128902 X03_30041741
X03_30153633 X03_33731899
X03_34078661 X03_36648091
X03_36667059 X03_37697237
X03_37768709
X03_40919093 X03_42873069
X03_42873199 X03_43627234
X03_46845215 X03_47217461
X03_47350251 X03_48065704
X03_48098337
X03_48108820 X03_48281520
X03_48531666 X03_49788273
X03_51230810
X03_53302873 X03_53975357
X03_55273192 X03_55273318
X03_55515539 X03_55529625
X03_55980345 X03_56023312
X03_56264085 X03_56279204
X03_56381055
X03_56510773 X03_56573136
X03_56639702 X03_56718661
X03_56879883
X03_56992482
X03_57264120
X03_57423317 X03_57678338
X03_57742172
X03_57963212
X03_57985730
X03_58386463 X03_58439037
X03_58503971
X03_58574377 X03_58654154
X03_58708187 X03_58754293
X03_58846611
X03_58952743 X03_59065711
X03_59073814
X03_59101782 X03_59108389
X03_59144298 X03_59174609
X03_59209883
X03_59247312 X03_59253811
X03_59297079
X03_59365879 X03_59433863
X03_59440883 X03_59619807
X03_59714430
X03_59874949 X03_59884625
X03_60150672 X03_60194202
X03_60220793
X03_60247504 X03_60288870
Firm3.1
Flw3.1
FW3.1
FW3.2
Leaf3.1
RIP3.1
RIP3.2
SSC3.1
Chr 3 [3]
200
X03_60392846 X03_60558507
X03_60621227 X03_60673779
X03_60821344
X03_60976463 X03_61011187
X03_61049092
X03_61194145 X03_61515277
X03_61695586 X03_61856514
X03_61912611 X03_62105459
X03_62140362
X03_62287203
X03_62529768
X03_62812925 X03_62883692
X03_62931750
X03_63049009
X03_63063888 X03_63224408
X03_63295128
X03_63463347 X03_63728255
X03_63790092
X03_63966105
X03_64093859 X03_64316094
X03_64579995
X03_64794911
FW3.2
FW3.3
RIP3.2
SSC3.1

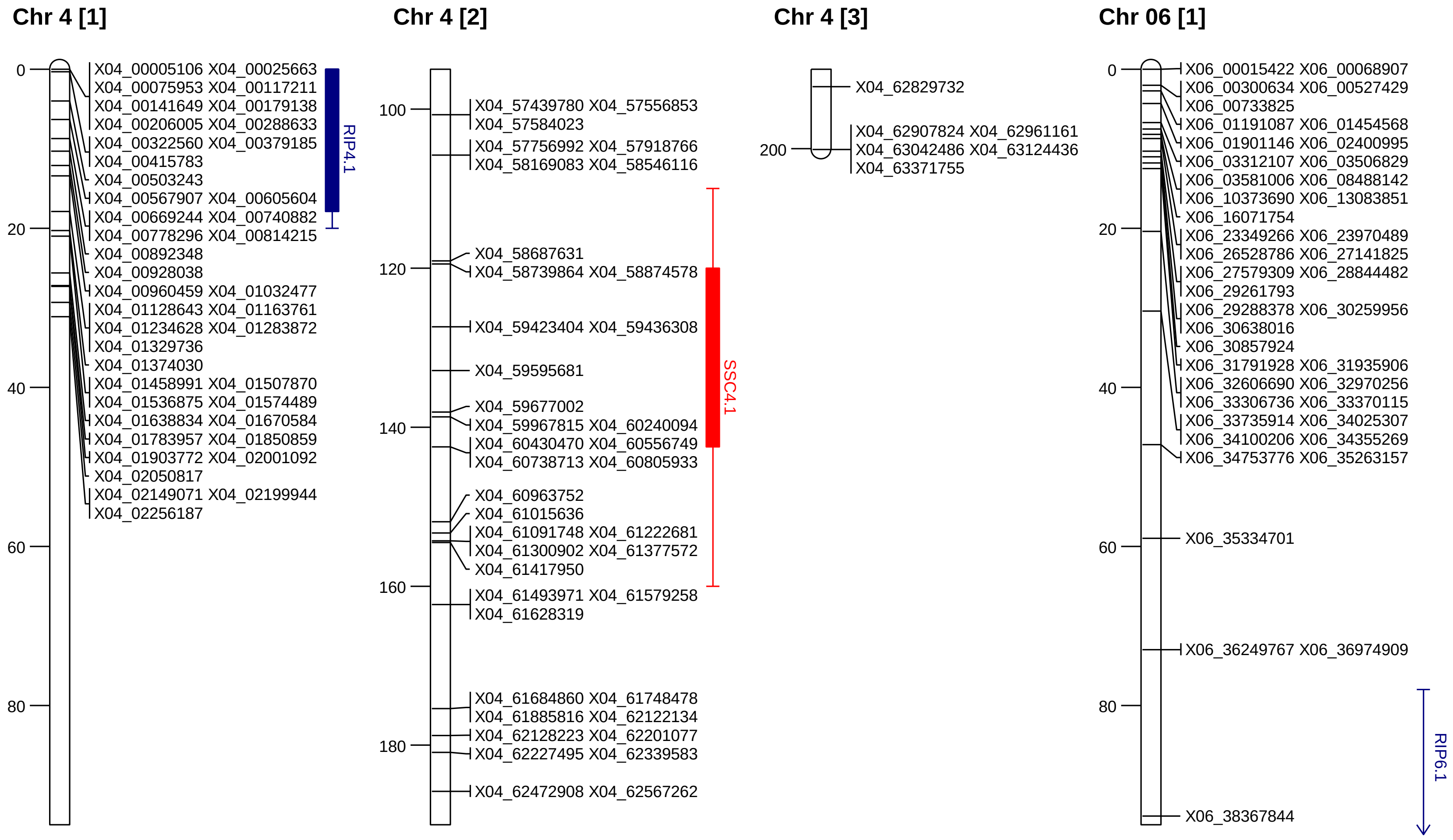
Chr 4 [1]
Chr 4 [2]
Chr 4 [3]
Chr 06 [1]
0
20
40
60
80
100
120
140
160
180
200
X04_00005106 X04_00025663
X04_00075953 X04_00117211
X04_00141649 X04_00179138
X04_00206005 X04_00288633
X04_00322560 X04_00379185
X04_00415783
X04_00503243
X04_00567907 X04_00605604
X04_00669244 X04_00740882
X04_00778296 X04_00814215
X04_00892348
X04_00928038
X04_00960459 X04_01032477
X04_01128643 X04_01163761
X04_01234628 X04_01283872
X04_01329736
X04_01374030
X04_01458991 X04_01507870
X04_01536875 X04_01574489
X04_01638834 X04_01670584
X04_01783957 X04_01850859
X04_01903772 X04_02001092
X04_02050817
X04_02149071 X04_02199944
X04_02256187
RIP4.1
X04_57439780 X04_57556853
X04_57584023
X04_57756992 X04_57918766
X04_58169083 X04_58546116
X04_58687631
X04_58739864 X04_58874578
X04_59423404 X04_59436308
X04_59595681
X04_59677002
X04_59967815 X04_60240094
X04_60430470 X04_60556749
X04_60738713 X04_60805933
X04_60963752
X04_61015636
X04_61091748 X04_61222681
X04_61300902 X04_61377572
X04_61417950
X04_61493971 X04_61579258
X04_61628319
X04_61684860 X04_61748478
X04_61885816 X04_62122134
X04_62128223 X04_62201077
X04_62227495 X04_62339583
X04_62472908 X04_62567262
SSC4.1
X04_62829732
X04_62907824 X04_62961161
X04_63042486 X04_63124436
X04_63371755
X06_00015422 X06_00068907
X06_00300634 X06_00527429
X06_00733825
X06_01191087 X06_01454568
X06_01901146 X06_02400995
X06_03312107 X06_03506829
X06_03581006 X06_08488142
X06_10373690 X06_13083851
X06_16071754
X06_23349266 X06_23970489
X06_26528786 X06_27141825
X06_27579309 X06_28844482
X06_29261793
X06_29288378 X06_30259956
X06_30638016
X06_30857924
X06_31791928 X06_31935906
X06_32606690 X06_32970256
X06_33306736 X06_33370115
X06_33735914 X06_34025307
X06_34100206 X06_34355269
X06_34753776 X06_35263157
X06_35334701
X06_36249767 X06_36974909
X06_38367844
RIP6.1

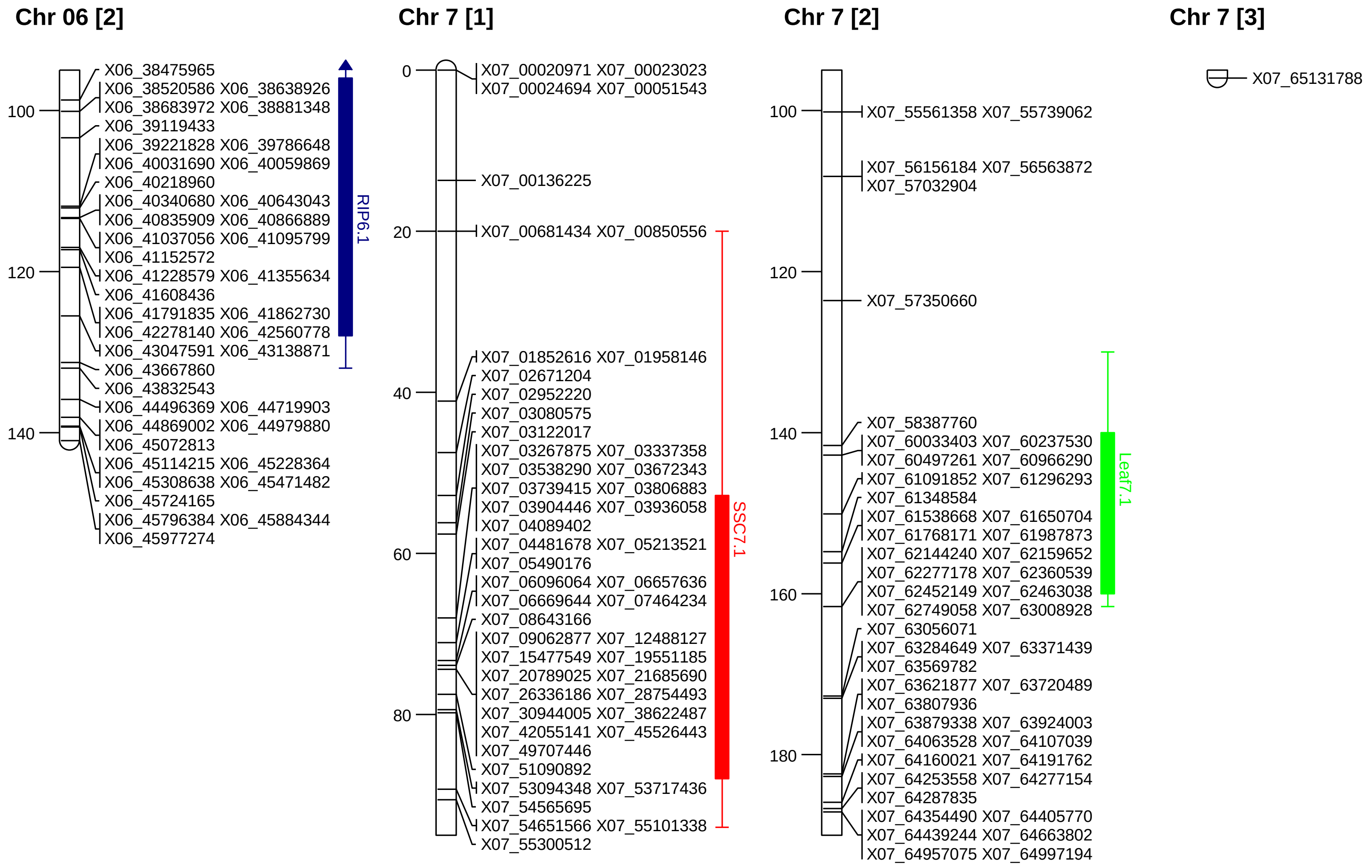
Chr 06 [2]
Chr 7 [1]
Chr 7 [2]
Chr 7 [3]
X06_38475965
X06_38520586 X06_38638926
X06_38683972 X06_38881348
X06_39119433
X06_39221828 X06_39786648
X06_40031690 X06_40059869
X06_40218960
X06_40340680 X06_40643043
X06_40835909 X06_40866889
X06_41037056 X06_41095799
X06_41152572
X06_41228579 X06_41355634
X06_41608436
X06_41791835 X06_41862730
X06_42278140 X06_42560778
X06_43047591 X06_43138871
X06_43667860
X06_43832543
X06_44496369 X06_44719903
X06_44869002 X06_44979880
X06_45072813
X06_45114215 X06_45228364
X06_45308638 X06_45471482
X06_45724165
X06_45796384 X06_45884344
X06_45977274
RIP6.1
X07_00020971 X07_00023023
X07_00024694 X07_00051543
X07_00136225
X07_00681434 X07_00850556
X07_01852616 X07_01958146
X07_02671204
X07_02952220
X07_03080575
X07_03122017
X07_03267875 X07_03337358
X07_03538290 X07_03672343
X07_03739415 X07_03806883
X07_03904446 X07_03936058
X07_04089402
X07_04481678 X07_05213521
X07_05490176
X07_06096064 X07_06657636
X07_06669644 X07_07464234
X07_08643166
X07_09062877 X07_12488127
X07_15477549 X07_19551185
X07_20789025 X07_21685690
X07_26336186 X07_28754493
X07_30944005 X07_38622487
X07_42055141 X07_45526443
X07_49707446
X07_51090892
X07_53094348 X07_53717436
X07_54565695
X07_54651566 X07_55101338
X07_55300512
SSC7.1
X07_55561358 X07_55739062
X07_56156184 X07_56563872
X07_57032904
X07_57350660
X07_58387760
X07_60033403 X07_60237530
X07_60497261 X07_60966290
X07_61091852 X07_61296293
X07_61348584
X07_61538668 X07_61650704
X07_61768171 X07_61987873
X07_62144240 X07_62159652
X07_62277178 X07_62360539
X07_62452149 X07_62463038
X07_62749058 X07_63008928
X07_63056071
X07_63284649 X07_63371439
X07_63569782
X07_63621877 X07_63720489
X07_63807936
X07_63879338 X07_63924003
X07_64063528 X07_64107039
X07_64160021 X07_64191762
X07_64253558 X07_64277154
X07_64287835
X07_64354490 X07_64405770
X07_64439244 X07_64663802
X07_64957075 X07_64997194
Leaf7.1
X07_65131788
0
20
40
60
80
100
120
140
160
180

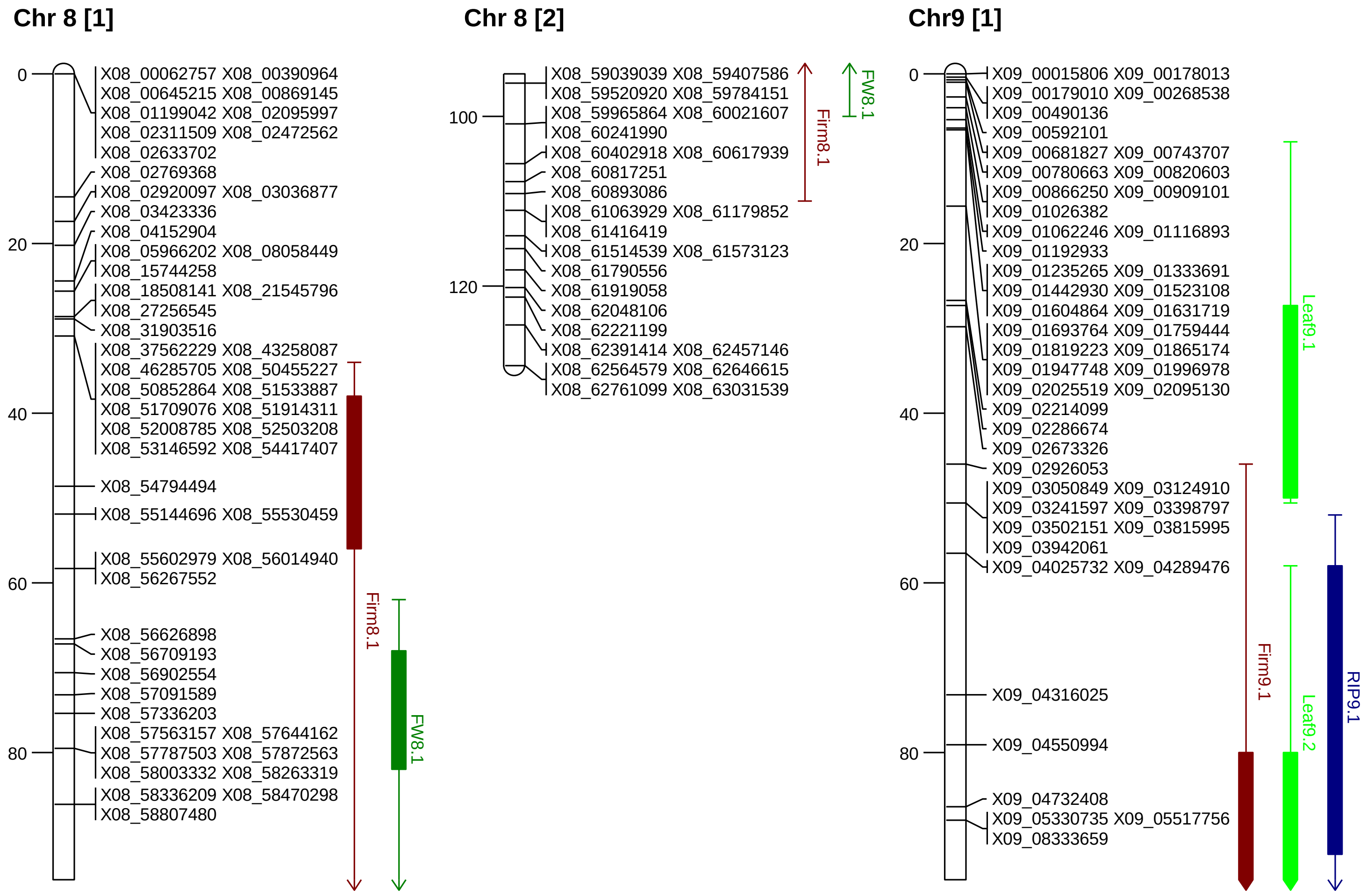
Chr 8 [1]
Chr 8 [2]
Chr9 [1]
0
20
40
60
80
100
120
X08_00062757 X08_00390964
X08_00645215 X08_00869145
X08_01199042 X08_02095997
X08_02311509 X08_02472562
X08_02633702
X08_02769368
X08_02920097 X08_03036877
X08_03423336
X08_04152904
X08_05966202 X08_08058449
X08_15744258
X08_18508141 X08_21545796
X08_27256545
X08_31903516
X08_37562229 X08_43258087
X08_46285705 X08_50455227
X08_50852864 X08_51533887
X08_51709076 X08_51914311
X08_52008785 X08_52503208
X08_53146592 X08_54417407
X08_54794494
X08_55144696 X08_55530459
X08_55602979 X08_56014940
X08_56267552
X08_56626898
X08_56709193
X08_56902554
X08_57091589
X08_57336203
X08_57563157 X08_57644162
X08_57787503 X08_57872563
X08_58003332 X08_58263319
X08_58336209 X08_58470298
X08_58807480
Firm8.1
FW8.1
X08_59039039 X08_59407586
X08_59520920 X08_59784151
X08_59965864 X08_60021607
X08_60241990
X08_60402918 X08_60617939
X08_60817251
X08_60893086
X08_61063929 X08_61179852
X08_61416419
X08_61514539 X08_61573123
X08_61790556
X08_61919058
X08_62048106
X08_62221199
X08_62391414 X08_62457146
X08_62564579 X08_62646615
X08_62761099 X08_63031539
FW8.1
Firm8.1
X09_00015806 X09_00178013
X09_00179010 X09_00268538
X09_00490136
X09_00592101
X09_00681827 X09_00743707
X09_00780663 X09_00820603
X09_00866250 X09_00909101
X09_01026382
X09_01062246 X09_01116893
X09_01192933
X09_01235265 X09_01333691
X09_01442930 X09_01523108
X09_01604864 X09_01631719
X09_01693764 X09_01759444
X09_01819223 X09_01865174
X09_01947748 X09_01996978
X09_02025519 X09_02095130
X09_02214099
X09_02286674
X09_02673326
X09_02926053
X09_03050849 X09_03124910
X09_03241597 X09_03398797
X09_03502151 X09_03815995
X09_03942061
X09_04025732 X09_04289476
X09_04316025
X09_04550994
X09_04732408
X09_05330735 X09_05517756
X09_08333659
Leaf9.1
Firm9.1
Leaf9.2
RIP9.1

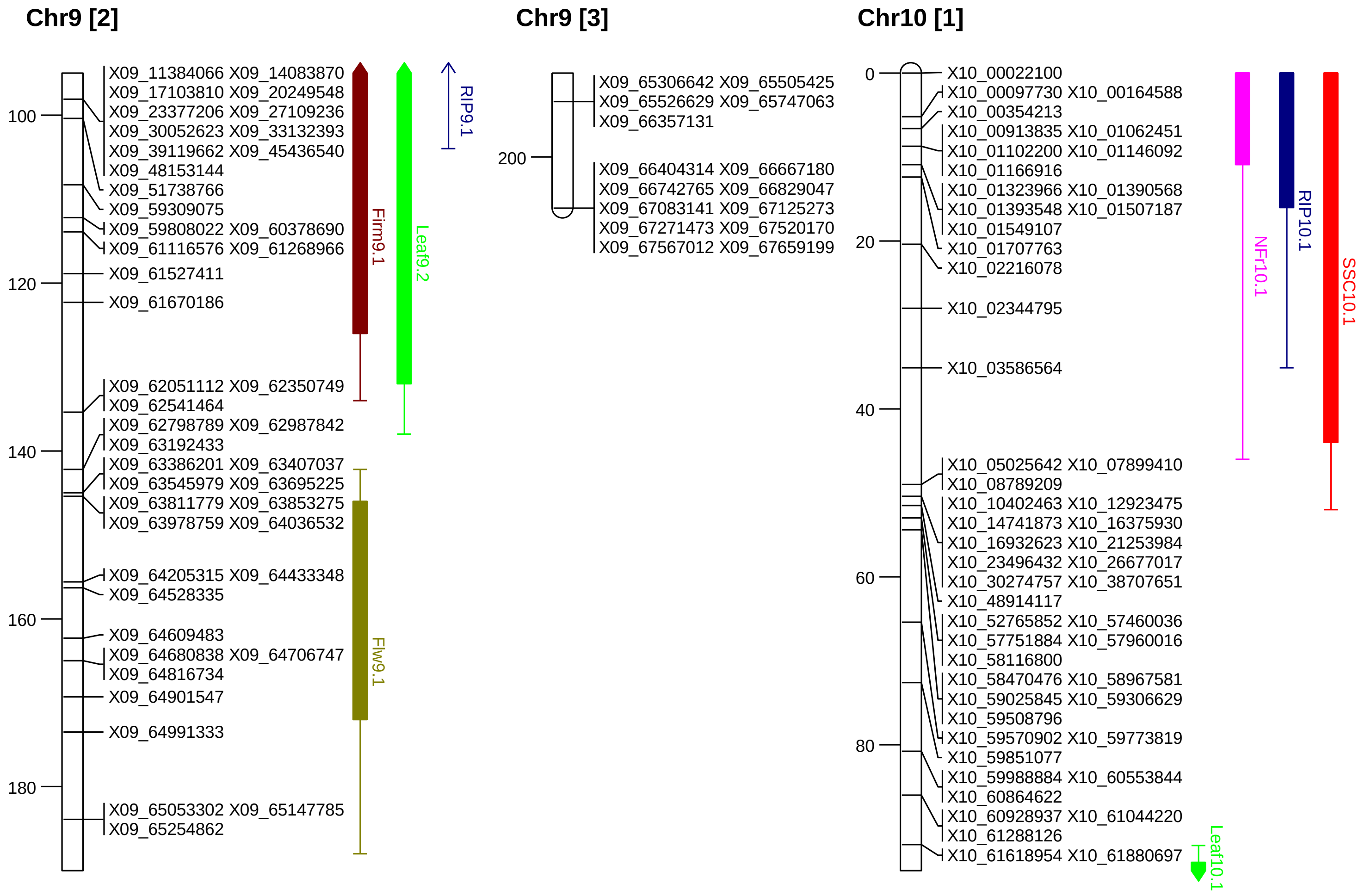
Chr9 [2]
X09_11384066 X09_14083870
X09_17103810 X09_20249548
X09_23377206 X09_27109236
X09_30052623 X09_33132393
X09_39119662 X09_45436540
X09_48153144
X09_51738766
X09_59309075
X09_59808022 X09_60378690
X09_61116576 X09_61268966
X09_61527411
X09_61670186
X09_62051112 X09_62350749
X09_62541464
X09_62798789 X09_62987842
X09_63192433
X09_63386201 X09_63407037
X09_63545979 X09_63695225
X09_63811779 X09_63853275
X09_63978759 X09_64036532
X09_64205315 X09_64433348
X09_64528335
X09_64609483
X09_64680838 X09_64706747
X09_64816734
X09_64901547
X09_64991333
X09_65053302 X09_65147785
X09_65254862
100
120
140
160
180
Firm9.1
Leaf9.2
RIP9.1
Flw9.1
Chr9 [3]
X09_65306642 X09_65505425
X09_65526629 X09_65747063
X09_66357131
200
X09_66404314 X09_66667180
X09_66742765 X09_66829047
X09_67083141 X09_67125273
X09_67271473 X09_67520170
X09_67567012 X09_67659199
Chr10 [1]
0
X10_00022100
X10_00097730 X10_00164588
X10_00354213
X10_00913835 X10_01062451
X10_01102200 X10_01146092
X10_01166916
X10_01323966 X10_01390568
X10_01393548 X10_01507187
X10_01549107
20
X10_01707763
X10_02216078
X10_02344795
X10_03586564
40
X10_05025642 X10_07899410
X10_08789209
X10_10402463 X10_12923475
X10_14741873 X10_16375930
X10_16932623 X10_21253984
X10_23496432 X10_26677017
60
X10_30274757 X10_38707651
X10_48914117
X10_52765852 X10_57460036
X10_57751884 X10_57960016
X10_58116800
X10_58470476 X10_58967581
X10_59025845 X10_59306629
X10_59508796
80
X10_59570902 X10_59773819
X10_59851077
X10_59988884 X10_60553844
X10_60864622
X10_60928937 X10_61044220
X10_61288126
X10_61618954 X10_61880697
NF10.1
RIP10.1
SSC10.1
Leaf10.1

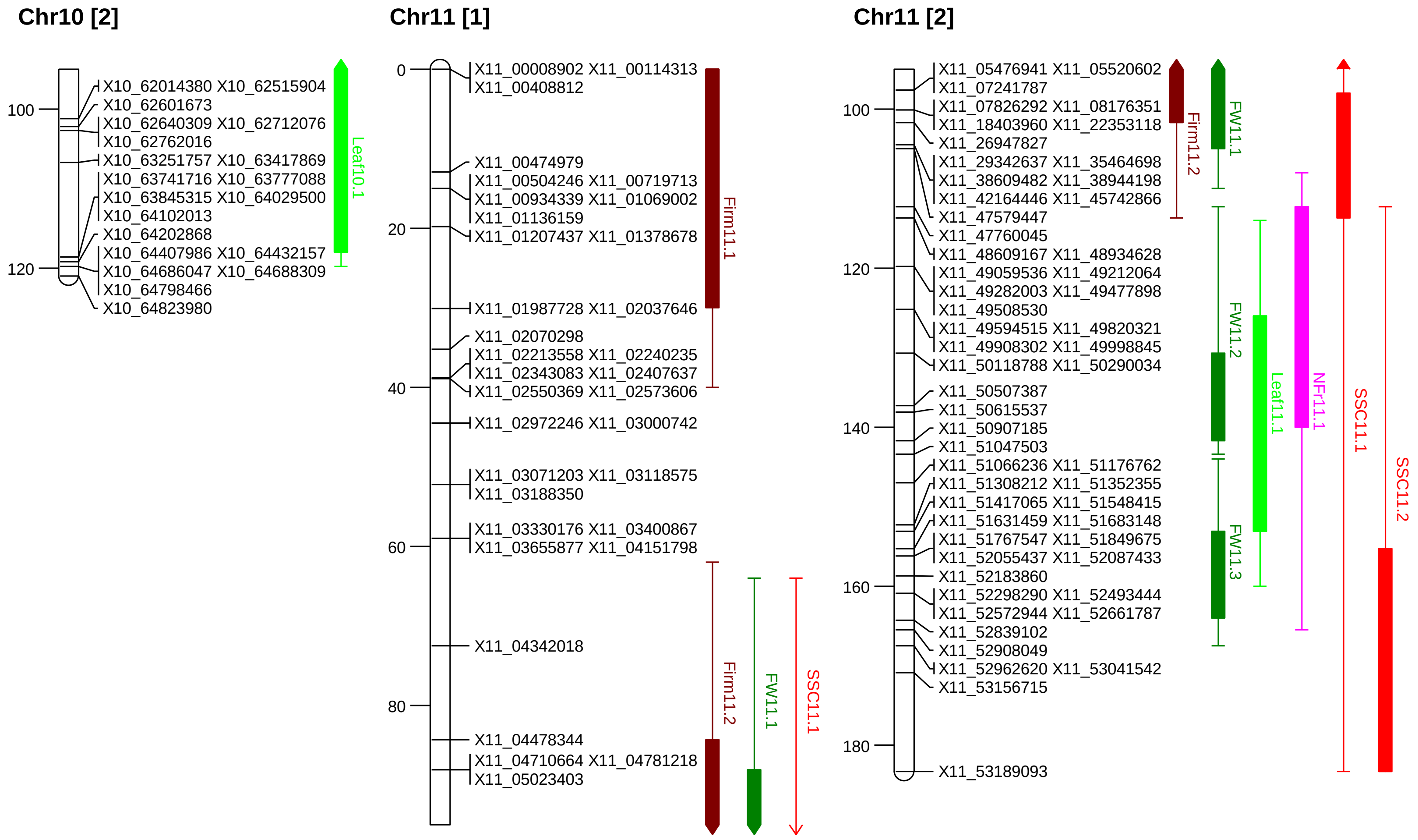
Chr10 [2]
Chr11 [1]
Chr11 [2]
100
120
X10_62014380 X10_62515904
X10_62601673
X10_62640309 X10_62712076
X10_62762016
X10_63251757 X10_63417869
X10_63741716 X10_63777088
X10_63845315 X10_64029500
X10_64102013
X10_64202868
X10_64407986 X10_64432157
X10_64686047 X10_64688309
X10_64798466
X10_64823980
Leaf10.1
0
20
40
60
80
X11_00008902 X11_00114313
X11_00408812
X11_00474979
X11_00504246 X11_00719713
X11_00934339 X11_01069002
X11_01136159
X11_01207437 X11_01378678
X11_01987728 X11_02037646
X11_02070298
X11_02213558 X11_02240235
X11_02343083 X11_02407637
X11_02550369 X11_02573606
X11_02972246 X11_03000742
X11_03071203 X11_03118575
X11_03188350
X11_03330176 X11_03400867
X11_03655877 X11_04151798
X11_04342018
X11_04478344
X11_04710664 X11_04781218
X11_05023403
Firm11.1
Firm11.2
FW11.1
SSC11.1
100
120
140
160
180
X11_05476941 X11_05520602
X11_07241787
X11_07826292 X11_08176351
X11_18403960 X11_22353118
X11_26947827
X11_29342637 X11_35464698
X11_38609482 X11_38944198
X11_42164446 X11_45742866
X11_47579447
X11_47760045
X11_48609167 X11_48934628
X11_49059536 X11_49212064
X11_49282003 X11_49477898
X11_49508530
X11_49594515 X11_49820321
X11_49908302 X11_49998845
X11_50118788 X11_50290034
X11_50507387
X11_50615537
X11_50907185
X11_51047503
X11_51066236 X11_51176762
X11_51308212 X11_51352355
X11_51417065 X11_51548415
X11_51631459 X11_51683148
X11_51767547 X11_51849675
X11_52055437 X11_52087433
X11_52183860
X11_52298290 X11_52493444
X11_52572944 X11_52661787
X11_52839102
X11_52908049
X11_52962620 X11_53041542
X11_53156715
X11_53189093
Firm11.2
FW11.1
FW11.2
FW11.3
Leaf11.1
NFr11.1
SSC11.1
SSC11.2

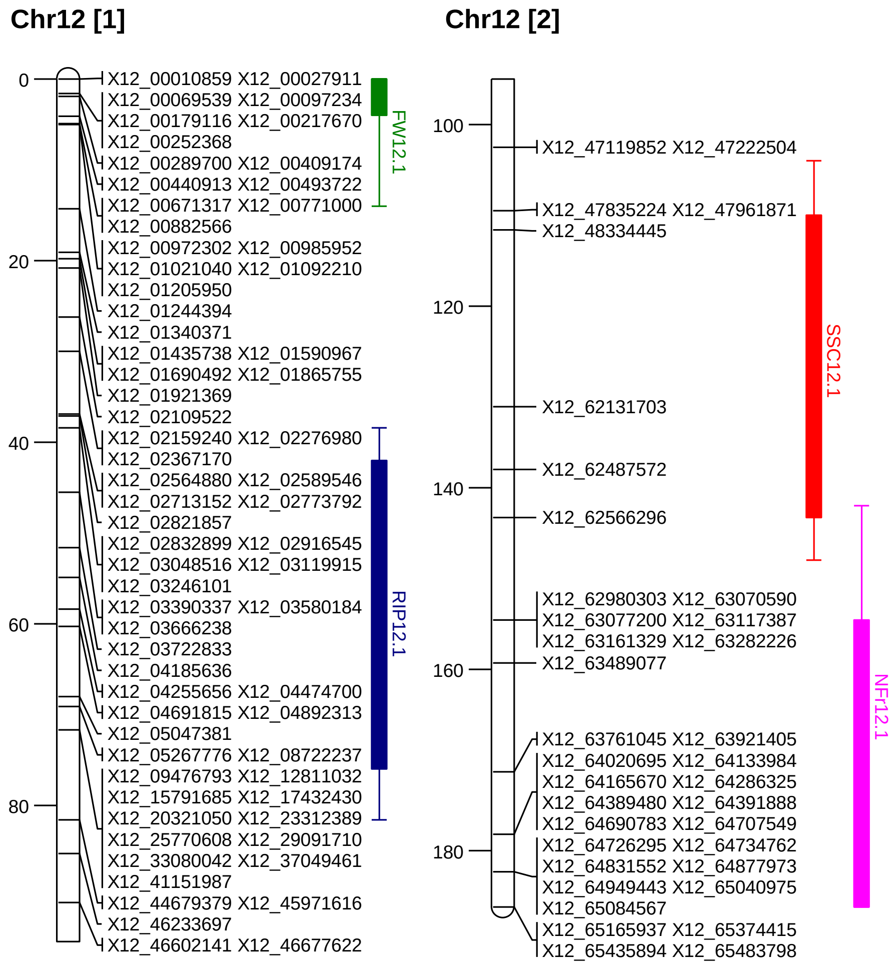
Chr12 [1]
Chr12 [2]
0
20
40
60
80
100
120
140
160
180
X12_00010859 X12_00027911
X12_00069539 X12_00097234
X12_00179116 X12_00217670
X12_00252368
X12_00289700 X12_00409174
X12_00440913 X12_00493722
X12_00671317 X12_00771000
X12_00882566
X12_00972302 X12_00985952
X12_01021040 X12_01092210
X12_01205950
X12_01244394
X12_01340371
X12_01435738 X12_01590967
X12_01690492 X12_01865755
X12_01921369
X12_02109522
X12_02159240 X12_02276980
X12_02367170
X12_02564880 X12_02589546
X12_02713152 X12_02773792
X12_02821857
X12_02832899 X12_02916545
X12_03048516 X12_03119915
X12_03246101
X12_03390337 X12_03580184
X12_03666238
X12_03722833
X12_04185636
X12_04255656 X12_04474700
X12_04691815 X12_04892313
X12_05047381
X12_05267776 X12_08722237
X12_09476793 X12_12811032
X12_15791685 X12_17432430
X12_20321050 X12_23312389
X12_25770608 X12_29091710
X12_33080042 X12_37049461
X12_41151987
X12_44679379 X12_45971616
X12_46233697
X12_46602141 X12_46677622
FW12.1
RIP12.1
X12_47119852 X12_47222504
X12_47835224 X12_47961871
X12_48334445
X12_62131703
X12_62487572
X12_62566296
X12_62980303 X12_63070590
X12_63077200 X12_63117387
X12_63161329 X12_63282226
X12_63489077
X12_63761045 X12_63921405
X12_64020695 X12_64133984
X12_64165670 X12_64286325
X12_64389480 X12_64391888
X12_64690783 X12_64707549
X12_64726295 X12_64734762
X12_64831552 X12_64877973
X12_64949443 X12_65040975
X12_65084567
X12_65165937 X12_65374415
X12_65435894 X12_65483798
SSC12.1
NFr12.1
